# Absence of Circadian Rhythm in Fecal Microbiota of Laying Hens under Common Light

**DOI:** 10.3390/ani11072065

**Published:** 2021-07-10

**Authors:** Yu Zhang, Lan Sun, Run Zhu, Shiyu Zhang, Shuo Liu, Yan Wang, Yinbao Wu, Xindi Liao, Jiandui Mi

**Affiliations:** 1 National Engineering Research Center for Breeding Swine Industry, College of Animal Science, South China Agriculture University, Guangzhou 510642, China; zhngyu1019@stu.scau.edu.cn (Y.Z.); lansun@163.com (L.S.); zhurun20203139145@stu.scau.edu.cn (R.Z.); zhangshiyu1119@126.com (S.Z.); lsjiayouya@126.com (S.L.); ywang@scau.edu.cn (Y.W.); wuyinbao@scau.edu.cn (Y.W.); 2 Ministry of Agriculture Key Laboratory of Tropical Agricultural Environment, South China Agricultural University, Guangzhou 510642, China; 3 Guangdong Provincial Key Lab of Agro-Animal Genomics and Molecular Breeding and Key Lab of Chicken Genetics, Breeding and Reproduction, Ministry of Agriculture, Guangzhou 510642, China

**Keywords:** circadian rhythm, feces, laying hen, light, microbiota

## Abstract

**Simple Summary:**

The circadian rhythm of gut microbiota is an important biological rhythm that plays a crucial role in host health. This study showed that the defecation time may be an important factor in the diversity, proportion, and functions of the feces microbial community. However, there was no circadian rhythm of microbial community assembly confirmed by JTK_Cycle analysis, which is a tool for identifying and characterizing oscillations and can efficiently identify and characterize cycling variables in large data sets. These results might suggest there was no obvious circadian rhythm of fecal microbiota in laying hens under common light.

**Abstract:**

The circadian rhythm of gut microbiota is an important biological rhythm that plays a crucial role in host health. However, few studies have determined the associations between the circadian rhythm and gut microbiota in laying hens. The present experiment investigated the circadian rhythm of fecal microbiota in laying hens. Feces samples were collected from 10 laying hens at nine different time points (06:00–12:00–18:00–00:00–06:00–12:00–18:00–00:00–06:00) to demonstrate the circadian rhythm of fecal microbiota. The results showed that the α and β diversity of the fecal microbiota fluctuated significantly at different time points. Beta nearest taxon index analysis suggested that assembly strategies of the abundant and rare amplicon sequence variant (ASV) sub-communities were different. Abundant ASVs preferred dispersal limitation (weak selection), and rare ASVs were randomly formed due to the “non-dominant” fractions. Highly robust fluctuations of fecal microbiota at the phylum level were found. For example, Firmicutes and Proteobacteria fluctuated inversely to each other, but the total ratio remained in a dynamic balance over 48 h. We identified that temporal dynamic changes had a significant effect on the relative abundance of the important bacteria in the feces microbial community using the random forest algorithm. Eight bacteria, *Ruminococcus gnavus*, *Faecalibacterium*, Ruminococcaceae, *Enterococcus cecorum*, Lachnospiraceae, *Clostridium*, *Clostridiales*, and *Megamonas*, showed significant changes over time. One unexpected finding was the fact that these eight bacteria belong to Firmicutes. The pathways showed significant fluctuation, including xenobiotic biodegradation and metabolism, carbohydrate metabolism, and amino acid metabolism, which were consistent with the metabolic functions of amino acids and carbohydrates from the feed. This study showed that the defecation time may be an important factor in the diversity, proportion, and functions of the feces microbial community. However, there was no circadian rhythm of microbial community assembly confirmed by JTK_Cycle analysis. These results might suggest there was no obvious circadian rhythm of fecal microbiota in laying hens under common light.

## 1. Introduction

The poultry industry is an important component of the world agricultural economy because it provides a vital source of protein for the human diet [1,2,3], and this proportion is constantly growing [4]. However, intensive laying hen operations are facing a series of problems under the general trend of “no resistance breeding”, such as a high incidence of fatty liver [5], abdominal fat deposition [6], poor eggshell quality [7], and serious pecking addiction [8,9]. There is an urgent need to ensure the overall and intestinal health of laying hens to maintain the sustainable development of the industry.

The intestinal microbial community is a highly complex and diverse ecosystem that plays a crucial role in regulating host metabolism, immune modulation, and many other key physiological pathways and maintaining the homeostasis and health of the host [10,11,12,13,14]. The diversity of the intestinal microbiota has become a potential indicator of overall host health [15]. Targeted regulation of the intestinal microbiota of laying hens has become a new direction to improve the above-mentioned problems in laying hens. Most organisms have a circadian rhythm in physiology and behavior, which are synchronized with the 24 h light/dark cycle of the earth [16]. Circadian rhythms are an important biological rhythm that synchronizes with the metabolism of the body [17]. Recent researchers showed an increased interest in the circadian rhythm of the intestinal microbiota. The normal intestinal micro-ecosystem maintains a certain circadian rhythm with rhythmic fluctuations in composition and function, and disturbances in the intestinal microbiota affect host health [18,19]. The circadian rhythm of bacteria was first found in *Cyanobacteria* cells, and their metabolism levels changed with changes in illumination time [20]. Notably, the relative abundance of greater than 15% of intestinal microbiota showed periodic fluctuations, including *Clostridium* and *Bacteroides* [21]. The extensive research on the intestinal microbiota showed that intestinal microbiota exhibited a 24 h cycle of the circadian rhythm. The circadian rhythm plays an important role in the host’s physiological metabolism, as the intestinal microbiota participate in host metabolism [22,23]. Adjusting the illumination time [21,24] and eating time of mice [25] could regulate the composition and function of the intestinal microbiota over 24 h.

Compared with other animals, laying hens are more sensitive to light, which is an important factor in circadian rhythm, and have a stronger biological rhythm. A suitable illumination scheme improves the production performance of laying hens and ensures the health of laying hens [26]. Newborn chicks had different intestinal microbiota communities when exposed to different light conditions (12/12 h light and dark (L/D) and 23/1 h L/D) [27]. A small fraction of microbiota oscillated with a significant detectable rhythm (based on JTK_Cycle) and fewer still oscillated with a 24 h rhythm, which was one of the earliest studies to show that the photoperiod could regulate the intestinal microbiota communities of newly hatched chicks [27]. However, there is currently a lack of more detailed and specific research on whether the intestinal microbiota of laying hens have a circadian rhythm. Therefore, the experiment was to confirm whether there was a circadian rhythm in the fecal microbiota of Hy-Line Gray laying hens.

## 2. Materials and Methods

### 2.1. Animals and Feeding

Given their widespread application in the poultry industry, Hy-Line Gray laying hens were selected for this study. The 30-week-old Hy-Line Gray laying hens used in this experiment were all fed under the same feeding environment and dietary conditions in a commercial hatchery (Peng Chang Co., Ltd., Shenzhen, China). There were 3 laying hens in each cage, and there was enough space to ensure that they can move freely. A total of 10 laying hens with similar weights (2.05 ± 0.03 kg) and laying rates (95 ± 0.50%) were randomly selected from different cages and marked as specific numbers 1–10 and had been kept in commercial hatchery all the time. At the same time, they still stayed in their original cages and did not move to other places. During the experiment, the laying hens received the same commercial feed ad libitum. The diet was based on corn and soybean meal and was designed to meet or exceed the energy requirement of the NRC (2012) (Additional file: Table S1). Clean drinking water was also always provided. The laying hens were kept in a henhouse maintained at 25 °C with a 16 h light (from 6 a.m. to 10 p.m.) and an 8 h dark management schedule, and the experiment was performed for 2 days. None of the laying hens required antibiotics during the sampling period. The diet, age, weight, and feeding environment of all laying hens were consistent during the experiment to minimize the possible impact of these factors.

### 2.2. Feces sample Collection

Fresh feces samples were collected at 06:00–12:00–18:00–00:00–06:00–12:00–18:00–00:00–06:00 (Zeitgeber times [ZT] 0–48) over 48 h at nine different time points. At each time point, feces samples were collected within 30 min, and a total of 90 fresh feces samples were obtained from ten laying hens. Sterile gloves were worn during feces sample collection, and the anus and surrounding area of the laying hens were cleaned with sterile water and cotton soaked with 75% ethanol to minimize the risk of contamination. The feces samples were collected manually in sterile tubes. After collection, the samples were immediately placed in liquid nitrogen and stored at −80 °C for further experiments.

### 2.3. DNA Extraction and 16S rRNA Amplification

Approximately 200 mg of feces sample was used for total DNA extraction using a QIAamp Power Fecal Pro DNA Kit (QIAGEN GmbH, QIAGEN Strasse 1, Hilden, Germany) following the manufacturer’s instructions. The DNA samples were stored at −20 °C until further use. The fecal microbiota composition profiles over time were determined using 16S rRNA gene sequencing. The V3-V4 hypervariable regions in DNA samples were amplified using 16S rRNA gene PCR and specific primers (Forward: 5′- GTGCCAGCMGCCGCGGTAA-3′ and Reverse: 5′-GGACTACHVGGGTWTCTAAT-3′) [28]. PCR amplifications were performed using 25 µL reaction mixtures containing 11 µL of PCR-grade water, 10 µL of 5′ PRIME HotMasterMix, 3 µL of DNA template, and 0.5 µL of each primer (initial concentration: 10 µM). PCR amplifications were performed using the following conditions: predenaturation at 94 °C for 5 min; 30 cycles of amplification, including denaturation at 94 °C for 30 s, annealing at 50 °C for 30 s and elongation at 72 °C for 30 s; final elongation at 72 °C for 10 min. To ensure the efficiency and accuracy of amplification, the quantitative and qualitative inspection of DNA was performed using a Qubit nucleic acid quantitative analyzer and agarose gel electrophoresis. Unqualified samples were eliminated for classification, which resulted in the analysis of 86 feces samples (06:00(*n* = 9)–12:00(*n* = 10)–18:00(*n* = 10)–00:00(*n* = 10)–06:00(*n* = 10)–12:00(*n* = 9)–18:00(*n* = 10)–00:00(*n* = 9)–06:00(*n* = 9)). The final products were sequenced using the Illumina HiSeq PE250 platform. PCR amplification and sequencing were performed by Novogene Co., Ltd. (Tianjin, China).

### 2.4. Sequence Analysis

Raw reads were obtained from the Illumina HiSeq PE250 platform. Raw reads were uploaded into Quantitative Insights into Microbial Ecology (QIIME2–2020.6) software and qualitatively trimmed [29]. The unqualified reads and sequences with poor splicing effects (<25 mass scores and >225 bp length) were filtered using quality monitoring to obtain clean reads. Trimmed sequences were clustered using the DADA2 method with amplicon sequence variant (ASV) levels [30]. The parameters of DADA2 were set at --p-trim-left-f 0, --p-trim-left-r 0, --p-trunc-len-f 200, and --p-trunc-len-r 200 and the reads with non-chimeric were range from 46, 014 to 87, 152 with average 75, 605. The rarity curves of 86 samples were stable, and the sampling depth was sufficient to describe the microbial community in the feces samples of laying hens (Additional file: Figure S1). All clean reads were compared with reference sequences to obtain the final mapped reads. Analysis of alpha diversity (Observed species, Chao 1, and Shannon indexes) was calculated in QIIME. For beta diversity analysis, principle coordinates analysis (PCoA) performed using the QIIME2 software was used to evaluate the difference in bacterial community structure. The functional prediction of the microbial community was based on the Phylogenetic Investigation of Communities by Reconstruction of Unobserved States (PICRUSt) [31] and comparisons in the Kyoto Encyclopedia of Genes and Genomes (KEGG) database [32].

### 2.5. Diurnal Analysis of Microbiome Data Sets and MNTD Taxonomic β-Diversity Metrics

For prediction of the circadian rhythm in fecal microbiota, we used the nonparametric test JTK_Cycle, with oscillations tested for a 24 h period length (Hughes et al., 2010). Significance was determined if the *p* values (ADJ.P) and Benjamini–Hochberg q values (BH. Q) were both less than 0.05. To evaluate the community assembly processes, the mean nearest taxon distance (MNTD) taxonomic β-diversity metrics (βNTI and Bray–Curtisbased Raup-Crick, RC_Bray_) was calculated as previously described [33]. |βNTI| > 2 means that the deterministic process was the main factor to influence the microbial community across all samples. In detail, a βNTI with a value of <−2 suggests homogeneous selection and >2 means variable selection. If |βNTI| < 2, the RC_Bray_ should be calculated: (1) RC_Bray_ > 0.95 means dispersal limitations, (2) RC_Bray_ < −0.95 reveals homogeneous dispersal, and (3) |RC_Bray_| < 0.95 indicates “non-dominant” fractions [34,35]. The interpretation degree of the difference in feces bacterial community structure was analyzed using a linear model, and the significance analysis was performed using the substitution test. *p*-values < 0.05 were considered statistically significant. The machine-learning method random forest (RF) was used to identify the top 30 genera and their functions at different times.

### 2.6. Construction of a Co-Occurrence Network

The cooccurrence mode of the dominant genera was constructed based on SparCC rank correlations of bacterial abundance on the network interface to understand the relationships between predominant genera in the feces samples. The effective cooccurrence events were based on strong and significant correlations between the predominant bacteria. Nodes in the network represented the predominant bacteria at the genus and family levels, and the edges indicated the relationship between these factors. The size of each node is proportional to its degree (the number of connections) in the dataset.

## 3. Results

### 3.1. Fecal Microbiota Structure in Laying Hens

**Figure 1:**
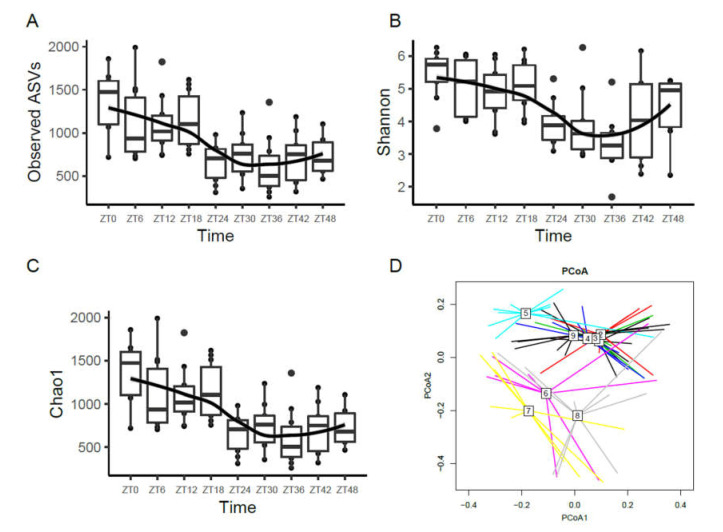
Changes in fecal microbiota diversity. (**A**) Bacterial alpha diversity determined by the Observed ASVs; (**B**) Bacterial alpha diversity determined by the Chao1 index; (**C**) Bacterial alpha diversity determined by the Shannon index; (**D**) PCoA of the fecal microbiota at each time point. The Black line represents the fitted trend line.

The time of defecation was the most significant factor to explain the differences between individuals in microbial community structure. The diversity of the feces microbial community of laying hens was evaluated using the Observed ASVs and Chao1 and Shannon indexes (Figure 1). According to the results, community diversity (Observed ASVs and Chao1 and Shannon indexes) fluctuated significantly at different time points. The Observed ASVs and Chao1 index of the feces microbial community of laying hens decreased gradually from 6:00 am on the first day to 12:00 on the second day but increased slowly from 12:00 midnight to 6:00 am on the third day (Figure 1A,B). Over time, the Shannon index also showed a trend of an initial decrease then a slow increase (Figure 1C). The alpha diversity of the fecal microbiota at 6:00 am on the first day was significantly higher than the other time points. To compare overall differences in the fecal microbiota composition of laying hens at nine different time points, we performed PCoA. The PCoA diagram shows that time points 1–4 are clustered together, time point 5 is separated, time points 6, 7, and 8 are further separated and clustered together, and time point 9 is clustered together with time points 1–4 (Figure 1D). Although some samples overlapped among the nine groups, there were moderate differences between the nine different groups in the microbiota. The results showed that the composition of the microbiota oscillated over time.

### 3.2. Fecal Microbiota Taxonomic Composition

**Figure 2:**
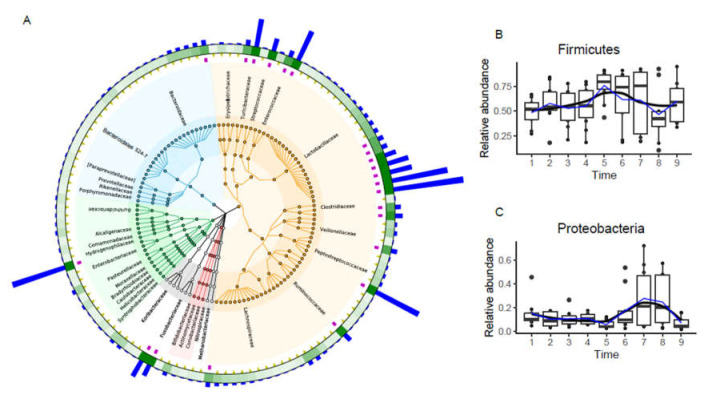
Different bacterial compositions based on the 16S rRNA data analysis. (**A**) GraPhlAn phylogenetic tree analysis of the fecal microbiota from the phylum to species level; (**B**,**C**) The temporal changes in the relative abundances of predominant bacteria (*Firmicutes* and *Proteobacteria*) at the phylum level. The black line represents the fitted trend line and the blue line represents the connecting line of the average value.

A GraPhlAn phylogenetic tree shows the relative abundance of taxonomic groups from the phylum to species level using the top 150 features (Figure 2A). There were six phylum-level taxonomic groups with high relative abundance, Firmicutes, Proteobacteria, Bacteroidetes, Acidobacteria, Fusobacteria, and Actinobacteria, and their average relative abundances accounted for 57.88, 14.11, 11.26, 6.23, 3.62, and 1.84% of the total sequences, respectively. Therefore, these groups were regarded as the predominant bacterial phyla because their mean relative abundances accounted for greater than 1% of the total sequences. Notably, Firmicutes and Proteobacteria were the most abundant bacteria in the feces microbial community of laying hens.

A total of 34 identified taxonomic groups were observed in the feces microbial community at the family level, and 12 of the most predominant bacterial populations were present, including Turicibacteraceae, Streptococcaceae, Enterococcaceae, Lactobacillaceae, Clostridiaceae, Veillonellaceae, Peptostreptococcaceae, Ruminococcaceae, Lachnospiraceae, Bacteroidaceae, Enterobacteriaceae, and Fusobacteriaceae. Their mean relative abundance exceeded 1% of the total sequences, and all these major bacterial families accounted for 43.76% of the total sequences in the fecal microbiota of laying hens. The remaining bacterial families, including Erysipelotrichaceae, Bacteroidales S24-7, Paraprevotellaceae, Prevotellaceae, Rikenellaceae, Porphyromonadaceae, Burkholderiaceae, Alcaligenaceae, Comamonadaceae, Hydrogenophilaceae, Pasteurellaceae, Moraxellaceae, Bradyrhizobiaceae, Caulobacteraceae, Helicobacteraceae, Syntrophobacteraceae, Koribacteraceae, Bifidobacteriaceae, Actinomycetaceae, Coriobacteriaceae, Nitrospiraceae, and Methanobacteriaceae, were considered low-abundance bacterial families because their sequences accounted for <1% of the total sequences and only 24.91% of the total sequences in the feces samples. Lactobacillaceae (7.95%) belongs to the Firmicutes phylum, which was the most abundant classification group in the feces community of laying hens and the most dominant family in the feces bacterial communities. The family-level taxonomic groups of the Firmicutes phylum were Turicibacteraceae, Streptococcaceae, Enterococcaceae, Lactobacillaceae, Clostridiaceae, Veillonellaceae, Peptostreptococcaceae, Ruminococcaceae, and Lachnospiraceae. Classified taxa in the Proteobacteria, Bacteroidetes, and Fusobacteria phyla at the family level were Bacteroidaceae, Enterobacteriaceae, and Fusobacteriaceae, respectively.

Further data analyses were performed to confirm whether the fecal microbiota showed circadian rhythm. Therefore, box and line maps were made for eight bacterial phyla of the fecal microbiota of laying hens at the phylum level to show the relative abundances of the predominant phyla. The results showed that the relative abundances of the two most dominant phyla had an obvious oscillation that oscillated in antiphase. Highly robust fluctuations of fecal microbiota at the phylum level were found. For instance, Firmicutes and Proteobacteria had opposite fluctuations (Figure 2B,C). The relative abundance of Firmicutes reached its peak and Proteobacteria reached its trough at 06:00 the next morning. The changing trends in the relative abundances of the other six predominant phyla did not fluctuate greatly (Additional file: Figure S2). In addition, JTK_Cycle analysis showed that eight predominant bacteria at phylum level showed no circadian rhythm (assessed as periods 24 h, JTK ADJ.P < 0.05 and BH. Q < 0.05) (Additional file: Table S2). Thus, the above results suggested eight predominant bacteria at the phylum level were no circadian rhythm.

**Figure 3:**
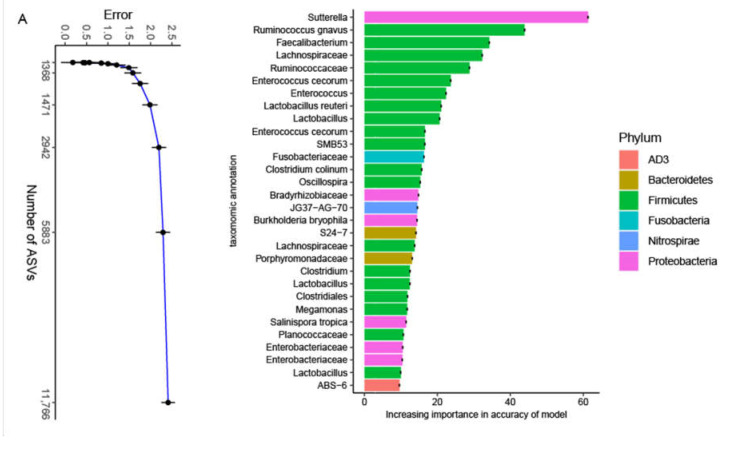

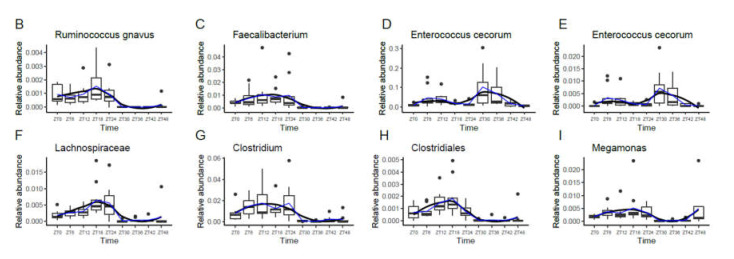
The most abundant bacteria were identified by random forest analysis. (**A**) Tracing the source of the top 30 most abundant bacteria in the fecal microbiota; (**B**–**I**) The temporal changes in the relative abundance of the eight most important bacteria. The black line represents the fitted trend line and the blue line represents the connecting line of the average value.

The 16S rRNA data of all feces samples were analyzed using the RF algorithm [36] to determine the most important ASVs, and the top 30 important ASVs were *Sutterella*, *Ruminococcus gnavus*, *Faecalibacterium*, Lachnospiraceae, Ruminococcaceae, *Enterococcus cecorum*, *Enterococcus*, *Lactobacillus reuteri*, *Lactobacillus*, Clostridiaceae SMB53, Fusobacteriaceae, *Clostridium colinum*, *Oscillospira*, Bradyrhizobiaceae, Nitrospiraceae JG37−AG−70, *Burkholderia bryophila*, Bacteroidales S24−7, Porphyromonadaceae, *Clostridium*, *Clostridiales*, *Megamonas*, *Salinispora tropica*, Planococcaceae, Enterobacteriaceae, and AD3 ABS−6. These bacteria were classified into the phyla AD3, Bacteroidetes, Firmicutes, Fusobacteria, Nitrospirae, and Proteobacteria (Figure 3A). AD3 ABS−6 was the taxa related to AD3 in the feces of laying hens with the lowest importance. Two ASVs, Bacteroidales S24−7 and Porphyromonadaceae, were related to Bacteroidetes. Firmicutes was the most abundant, including *Ruminococcus gnavus*, *Faecalibacterium*, Lachnospiraceae, Ruminococcaceae, *Enterococcus cecorum*, *Enterococcus*, *Lactobacillus reuteri*, *Lactobacillus*, Clostridiaceae SMB53, *Clostridium colinum*, *Oscillospira*, *Clostridium*, *Clostridiales*, *Megamonas*, and Planococcaceae. Fusobacteria contains Fusobacteriaceae, and Nitrospirae contains Nitrospiraceae JG37−AG−70. Five ASVs, including *Sutterella*, Bradyrhizobiaceae, *Burkholderia bryophila*, *Salinispora tropica*, and Enterobacteriaceae, were related to Proteobacteria, and *Sutterella* had the highest importance. This result was a rather remarkable outcome and showed that eight ASVs, *Ruminococcus gnavus*, *Faecalibacterium*, *Enterococcus cecorum*, Ruminococcaceae, Lachnospiraceae, *Clostridium*, *Clostridiales*, and *Megamonas*, exhibited an oscillation of a steady rise initially then a slight decrease in relative abundance (Figure 3B–I). We found that the relative abundances of these bacteria, including *Ruminococcus gnavus*, *Faecalibacterium*, Lachnospiraceae, *Clostridiales*, and *Megamonas*, reached their peak at time point 4, while the peaks of other bacteria, including *Enterococcus cecorum*, Ruminococcaceae, and *Clostridium*, were delayed. The remaining ASVs remained in a stable state (Additional file: Figure S3). In addition, JTK_Cycle analysis showed that the overwhelming majority ASVs had no circadian rhythm, except *Enterococcus cecorum* (Additional file: Table S3). Although the composition of the microbiota was quite different, the common core bacterial community in the feces may play an important role. 

### 3.3. Turnover of the Total, Abundant, and Rare of ASV Fractions

**Figure 4:**
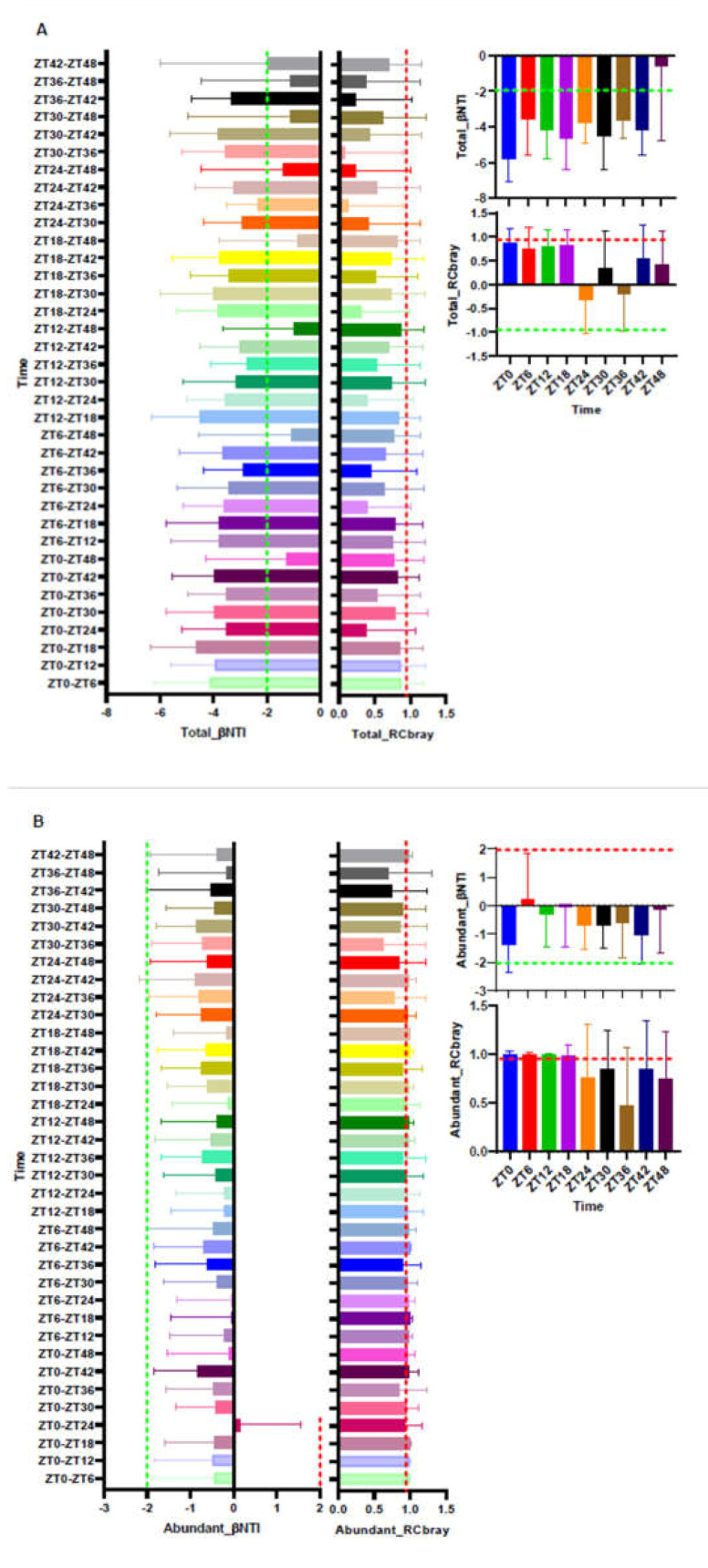

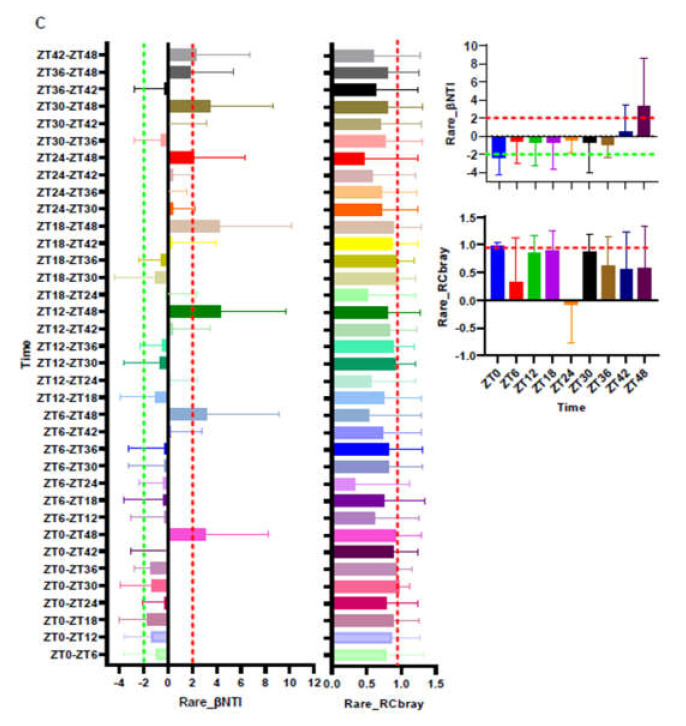
The Beta Nearest Taxon Index (βNTI) of the total (**A**), abundant (**B**), and rare (**C**) bacterial communities and the Raup–Crick metric based on relative abundances of the ASVs (RC_Bray_). The relative abundance of ASVs ≥ 1% and <0.1% were assigned as abundant ASVs and rare ASVs, respectively. Red (0.95 or 2) and green (−0.95 or −2) lines represent critical line values.

We calculated βNTI and RC_Bray_ to determine the assembly processes driving the circadian rhythm of microbial community composition. Whether for intra-group samples or inter-group samples, the most βNTI values between different samples were <−2 in the total ASVs, except the ZT48 time point, which suggests a deterministic process, i.e., homogeneous selection played a key role in shaping the microbial composition in this study. Conversely, in the abundant and rare ASV fractions, most βNTI values were between −2 and 2, which indicates that the stochastic process was important, except the ZT48 at rare ASVs was >2 (indicates variable selection). The RC_Bray_ values of the microbial communities in the abundant ASVs were >0.95, which reflects that the dispersal limitation (weak selection) dominantly determined the microbial community. However, the RC_Bray_ values of the microbial communities in the rare ASVs were <0.95, which indicates the “non-dominant” fractions (Figure 4).

### 3.4. Predicted Molecular Functions of Fecal Microbiota

**Figure 5:**
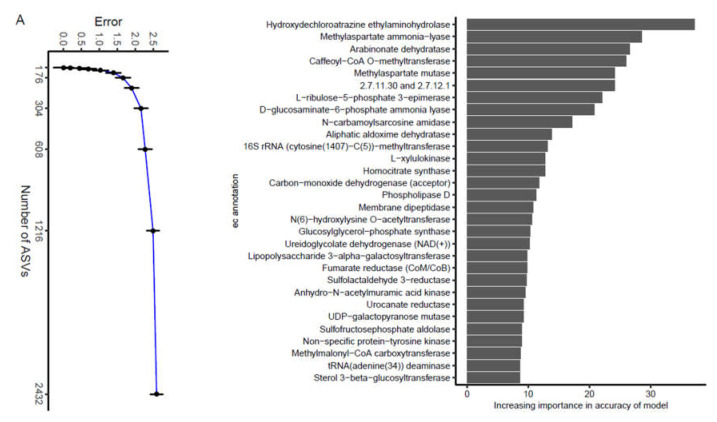

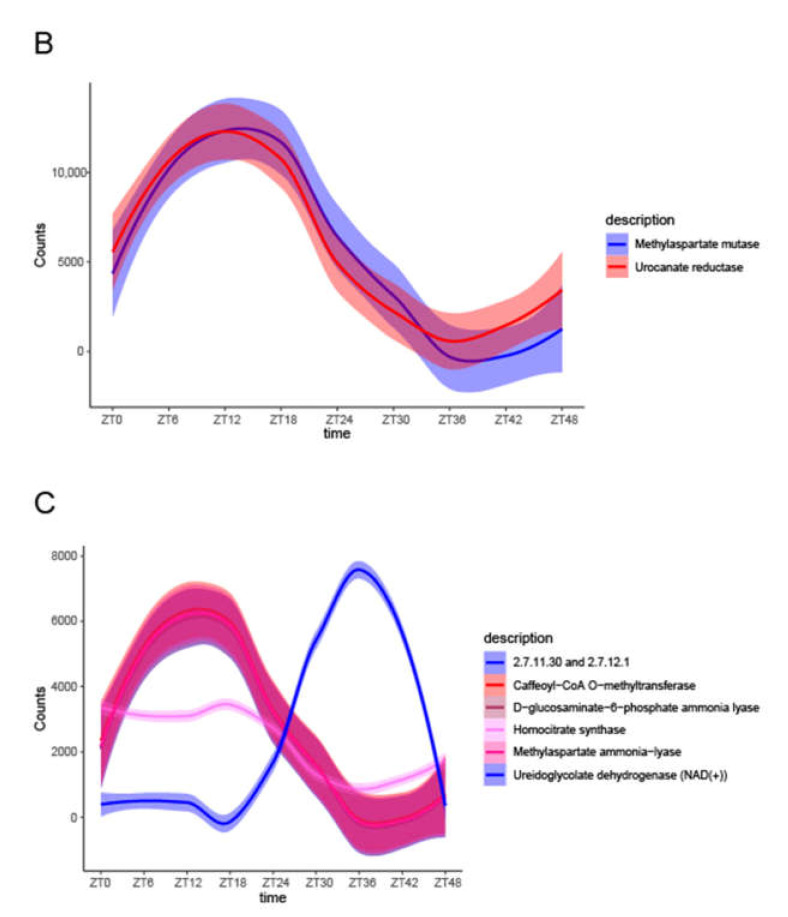
The most abundant enzymes were identified by random forest analysis. (**A**) Tracing the source of the top 30 most abundant enzymes in the fecal microbiota; (**B**,**C**) The temporal changes in the contributing enzymes.

We analyzed the composition of enzymes at level 3, and the top 30 important enzymes were selected and ranked according to importance from highest to lowest in the feces samples (Figure 5A). These profiles revealed that all enzymes at level 3 were related to metabolism. It showed that hydroxydechloroatrazine ethylaminohydrolase was the most predominant enzyme related to xenobiotic biodegradation and metabolism at level 2. Carbohydrate metabolism was related to eight enzymes at level 2: methylaspartate ammonia-lyase, methylaspartate mutase, L-ribulose-5-phosphate 3-epimerase, D-glucosaminate-6-phosphate ammonia lyase, L-xylulokinase, homocitrate synthase, UDP-galactopyranose mutase, and methylmalonyl-CoA carboxytransferase. N−carbamoylsarcosine amidase, homocitrate synthase and urocanate reductase were related to amino acid metabolism. Methylaspartate mutase, urocanate reductase, caffeoyl−CoA O−methyltransferase, D-glucosaminate-6-phosphate ammonia lyase, and methylaspartate ammonia-lyase showed significant changes over time (Figure 5B, C). There was no significant circadian rhythm in the remaining enzymes (Additional file: Figure S4). In addition, JTK_Cycle analysis showed that most enzymes showed no circadian rhythm, except Anhydro-N-acetylmuramic acid kinase and Fumarate reductase (CoM/CoB) (Additional file: Table S4). Further data analyses selected the related microbiota according to their contribution to these 30 enzymes (Additional file: Figure S5). We found that Fusobacteriaceae, Lactobacillaceae, and Enterobacteriaceae had major contributions to these enzymes. Burkholderiaceae, Clostridiaceae, Bradyrhizobiaceae, and Veillonellaceae were also involved in the contribution to these enzymes.

**Figure 6:**
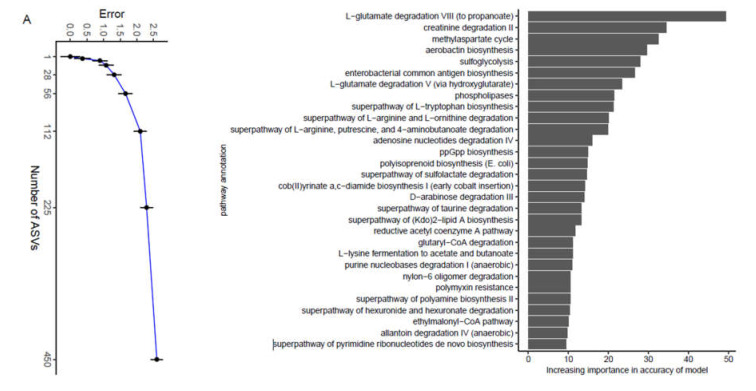

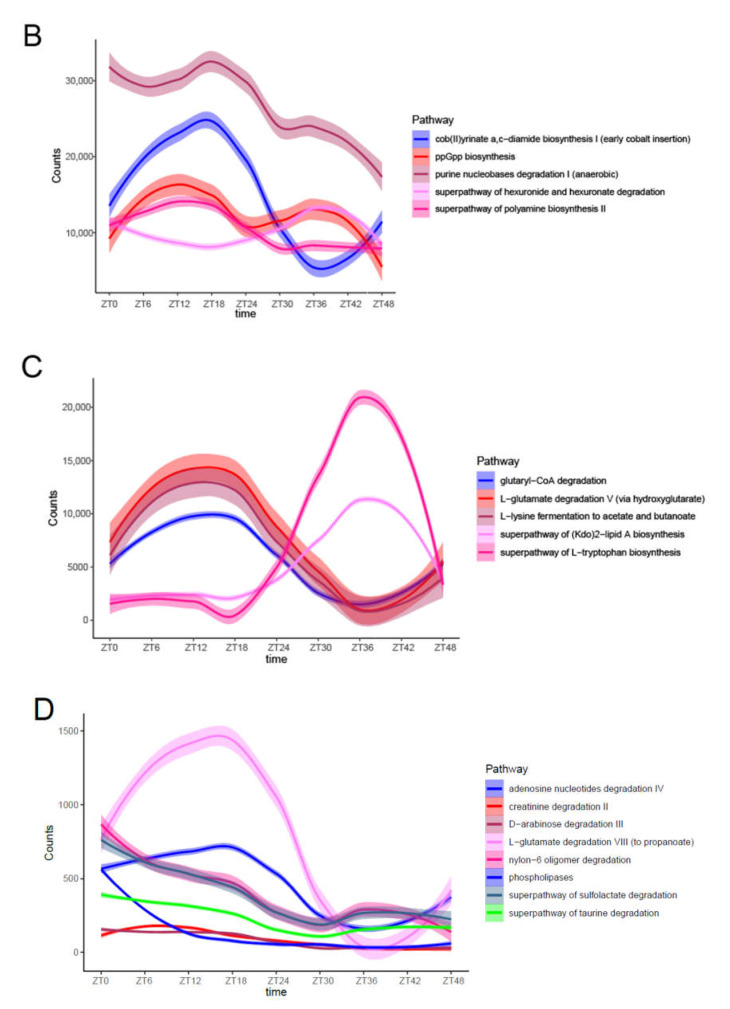
The important bacterial functions were identified by random forest analysis. (**A**) Tracing the source of the top 30 most important bacterial functions in the fecal microbiota; (**B**–**D**) The temporal changes in the contributing bacterial functions.

The differential abundance of the fecal microbiota created different functions of the microbiota. To understand the development of the functions of the feces microbial community over time, the Matacyc pathway compositions of the feces microbial community were predicted using PICRUSt based on the 16S rRNA data (Figure 6A). The predictable functions were sorted according to importance from highest to lowest. The 30 most symbolic Matacyc pathways that had been annotated at level 3 were identified in the feces samples, including L−glutamate degradation VIII (to propanoate), creatinine degradation II, methylaspartate cycle, aerobactin biosynthesis, sulfoglycolysis, enterobacterial common antigen biosynthesis, L−glutamate degradation V (via hydroxyglutarate), phospholipases, superpathway of L−tryptophan biosynthesis, superpathway of L−arginine and L−ornithine degradation, superpathway of L−arginine, putrescine, and 4−aminobutanoate degradation, adenosine nucleotides degradation IV, ppGpp biosynthesis, polyisoprenoid biosynthesis (*Escherichia coli*), superpathway of sulfolactate degradation, cob(II)yrinate a,c−diamide biosynthesis I (early cobalt insertion), D−arabinose degradation III, superpathway of taurine degradation, superpathway of (Kdo)2−lipid A biosynthesis, reductive acetyl coenzyme A pathway, glutaryl−CoA degradation, L−lysine fermentation to acetate and butanoate, purine nucleobases degradation I (anaerobic), nylon−6 oligomer degradation, polymyxin resistance, superpathway of polyamine biosynthesis II, superpathway of hexuronide and hexuronate degradation, ethylmalonyl−CoA pathway, allantoin degradation IV (anaerobic), and superpathway of pyrimidine ribonucleotides de novo biosynthesis (Figure 6A). Pathways involved in cob (II)yrinate a, c−diamide biosynthesis I (early cobalt insertion) was among the oscillating microbiota functions (Figure 6B). The functionalities that showed significant changes over time and included major pathways, such as glutaryl−CoA degradation, L−glutamate degradation V (via hydroxyglutarate), L−lysine fermentation to acetate (Figure 6C), and butanoate and L−glutamate degradation VIII (to propanoate) (Figure 6D), as exemplified by cob (II)yrinate a, c−diamide biosynthesis I (early cobalt insertion). There was no obvious rhythmic oscillation in the remaining pathways (Additional file: Figure S5). Taken together, these results suggested an association between the functionalities of the fecal microbiota and the passage of time. In addition, JTK_Cycle analysis showed that most pathways showed no circadian rhythm (Additional file: Table S5).

### 3.5. Cooccurrence Networks of Feces Bacteria

**Figure 7:**
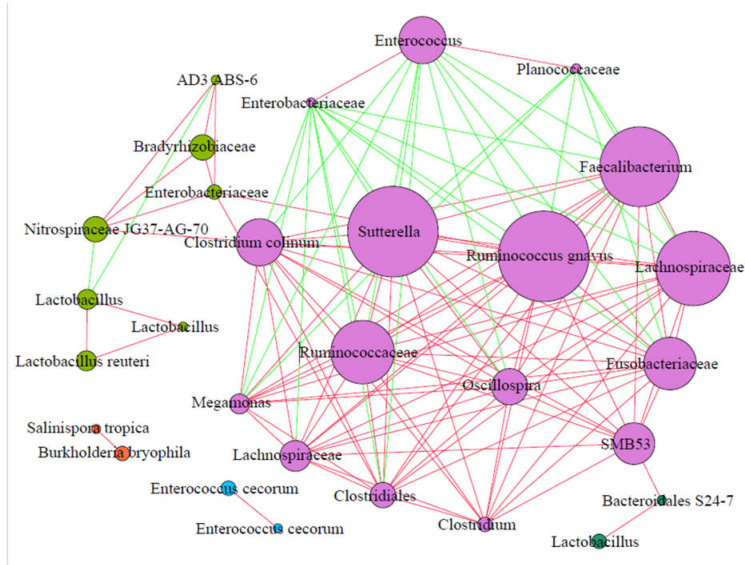
Interaction network of co-occurring important genera within feces bacteria in laying hens (*n* = 86). The node represents the predominant genera in laying hens’ feces, the size of each node represents the relative abundance, and the nodes are colored based on module structure. The edges represent negative (red) or positive (green) correlations of two connected nodes.

To identify the potential interactions between the fecal microbiota, correlative network analysis was performed for a feces bacterial community based on strong and significant correlations (Spearman’s *r*_s_ < −0.5 or *r*_s_ > 0.5, *p* < 0.01) (Figure 7). We performed a correlation analysis of the 30 ASVs identified earlier and found significant positive or negative correlations between these ASVs. The co-correlative network of the fecal microbiota consisted of 30 nodes (important bacteria). Five clusters (modules) were identified with high credibility in the bacterial correlative network in feces. *Sutterella* had positive correlations with *Faecalibacterium*, *Ruminococcus gnavus*, Lachnospiraceae, Fusobacteriaceae, *Oscillospira*, Clostridiaceae SMB53, *Clostridium*, *Clostridiales*, Ruminococcaceae, *Megamonas*, *Clostridium colinum*, and Nitrospiraceae JG37−AG−70. However, *Sutterella* had negative correlations with Enterobacteriaceae, *Enterococcus*, and Planococcaceae. *Enterococcus* had negative correlations with *Faecalibacterium*, Lachnospiraceae, Fusobacteriaceae, *Ruminococcus gnavus*, *Oscillospira*, *Sutterella*, Ruminococcaceae, *Clostridiales*, *Clostridium colinum*, and *Megamonas* but positive correlations with Enterobacteriaceae and Planococcaceae. The relative abundance of *Lactobacillus* showed positive correlations with *Lactobacillus reuteri* but negative correlations with Nitrospiraceae JG37−AG−70 and AD3 ABS−6 (Figure 7).

## 4. Discussion

The present study demonstrated the circadian rhythm of the fecal microbiota. We try to describe the phenomenon of a 24 h circadian rhythm of the fecal microbiota in laying hens using serial sampling for 48 h. However, there was no obvious circadian rhythm of fecal microbiota in laying hens in our research result under common lighting program (16L:8D). In a similar study, only a small fraction of microbiota of chicken oscillated with a circadian rhythm under a lighting program (12L:12D), while the microbiota of chicken did not oscillate in a 24 h rhythm under a lighting program (23L:1D) [27].

Alpha and beta diversity analyses indicated that temporal dynamic changes played an important role in the composition of the feces microbial community. These results corroborate the findings of a large amount of previous work on circadian variations of the relative abundances in the fecal microbiota in mouse stool samples [21,24,25,37]. The diversity of the fecal microbiota indicated that the microbial community may overlap partially, but most were scattered. The difference of fecal microbiota composition at different time points may be caused by the circadian rhythm of the host and the influence of the feeding cycle on the circadian rhythm.

We identified two main phyla in the fecal microbiota, Firmicutes and Proteobacteria, which is generally similar to previous results [38]. The relative abundance of these two bacteria had obvious oscillations that fluctuated inversely to each other over 48 h, but the total ratio remained in a dynamic balance, which may indicate a competitive relationship between these bacteria. The same oscillations in the relative abundance of Firmicutes also appeared in our results [21,25,37]. However, the findings were contrary to previous studies on the fecal microbiota of mouse stool samples, which found no circadian rhythm in the relative abundance of Proteobacteria [37]. This difference may be related to the variety of diets of the experimental animals. The 30 most important ASVs were selected using an RF model, and we found that temporal dynamic changes had a significant effect on the relative abundance of the important ASVs in the feces microbial community, among which eight ASVs, *Ruminococcus gnavus*, *Faecalibacterium*, Ruminococcaceae, *Enterococcus cecorum*, Lachnospiraceae, *Clostridium*, *Clostridiales*, and *Megamonas*, showed significant changes over time. One unexpected finding was the fact that all these eight ASVs belonged to Firmicutes. The microbiota profile appeared to favor butyrate production, potentially through increases in the members of Lachospiraceae, *Faecalibacterium,* and Ruminococcaceae [15,39]. The abundance of *Faecalibacterium*, Lachnospiraceae, and Ruminococcaceae reached their peak at the same time point 5(ZT24).

Our study found that the total ASVs was primarily driven by a deterministic process, and a stochastic process primarily dominated the abundant and rare ASVs. We also found that the rare ASVs were more ubiquitous than the abundant ASVs, which indicates that the abundant ASVs were driven by the time of feces excretion. This result may be explained by the fact that rare ASVs occupied a favorable position, competitively used a broader array of resources than the abundant ASVs, and effectively adapted to the environment [35]. Another possible explanation for this result was the βNTI and RC_Bray_ values, which reflected that the abundant ASV fraction was driven by a weaker selection compared to the rare ASV fraction, which was the “non-dominant” fractions. This result may be due to the lower competition capacity and growth rate of the abundant ASVs, which were limited in the environmental breadths [40].

The characteristics of the microbiota include its potential metabolic function and the enzymes involved in physiological metabolism. The potential functions of the fecal microbiota were determined using PICRUSt based on 16S rRNA data. The results showed that the most abundant functions, including xenobiotic biodegradation and metabolism, carbohydrate metabolism, and amino acid metabolism, were consistent with the metabolic functions of amino acids and carbohydrates, which are necessary for microbiota survival. Although the enzymes involved in the reaction had a significant effect on the most important microbiota functions at level 3, some of these enzymes gained significant fluctuation, including methylaspartate mutase, urocanate reductase, caffeoyl−CoA O−methyltransferase, D-glucosaminate-6-phosphate ammonia-lyase, and methylaspartate ammonia-lyase. Further analysis identified the related bacteria that contributed the most to these enzymes. Fusobacteriaceae, Lactobacillaceae, and Enterobacteriaceae had a major contribution and were the most predominant bacteria at the family level in the fecal microbiota. Prediction of the potential function of the fecal microbiota showed that cob(II)yrinate a, c−diamide biosynthesis I (early cobalt insertion), glutaryl−CoA degradation, L−glutamate degradation V (via hydroxyglutarate), L−lysine fermentation to acetate, and butanoate and L−glutamate degradation VIII (to propanoate) gained temporal changes in bacterial functions, which showed significant fluctuation upon physiological metabolism. The only reported glutaric acid catabolism was the dehydrogenation pathway of glutaryl-CoA [41]. Glutamate is a neurotransmitter that may be released from neurons or the gut microbiota [42], and its abundant presence is consistent with gut physiology and inflammation [43]. Its degradation may slow the inflammatory response. Most of these rhythmic pathways involve the degradation of amino acids and the production of short-chain fatty acids (SCFAs), such as acetic acid and propionic acid. The daily change in feces SCFA concentration was consistent with the rhythm of FFAR2/3 expression in the colonic muscularis [44]. SCFAs produced by microbiota fermentation may play an important role in maintaining circadian rhythm.

Our network analysis demonstrated that *Sutterella, Faecalibacterium*, *Ruminococcus gnavus*, Fusobacteriaceae, *Oscillospira*, Clostridiaceae SMB53, *Clostridium*, *Clostridiales*, Ruminococcaceae, Lachnospiraceae, *Megamonas*, *Clostridium colinum*, and Nitrospiraceae JG37−AG−70 had a positive correlation, potentially because all these bacteria are SCFA-producing strains. *Sutterella* was the most important ASV in the fecal microbiota in our results. However, previous studies concluded that *Sutterella* species were possible pro-inflammatory agents [45]. *Lactobacillus* spp. have various known benefits for metabolism and intestinal health, from antimicrobial to probiotic activity [46,47]. The relative abundance of Nitrospiraceae JG37−AG−70 negatively correlated with *Lactobacillus* abundance but positively correlated with *Clostridium* abundance, which is consistent with a previous report that *Lactobacillus* was depleted when pathogens, such as *Clostridium*, were enriched [48]. Multiple studies demonstrated that intestinal inflammation was frequently accompanied by an imbalanced microbial community, which is often characterized by a relative increase in facultative anaerobic Enterobacteriaceae [49]. The ubiquitous Gram-positive bacterium *Enterococcus* is a symbiotic bacterium of the intestinal tract, but it may cause serious opportunistic infections [50,51,52]. *Salinispora tropica*, *Burkholderia bryophila*, and *Enterococcus cecorum* were the important bacteria in the fecal microbiota, but they did not play an important role in the network and were completely independent of the outside two clusters. These important bacteria may not be functional in the network because of the high diversity of the fecal microbiota.

It is very important for laying hens to maintain intestinal health. As “the second genome” of the host, intestinal microbiota plays an important role in the health of the host and intestine, and its composition and function are influenced by many factors, such as variety, intestinal segment, feeding method, age, environment, and diet [53,54,55,56]. Previous studies showed that the circadian rhythm disorder of intestinal microbiota affected the biological clock and metabolic disorder of hosts and caused a series of diseases [25,57,58]. Therefore, maintenance of the rhythmic oscillations of the intestinal microbiota of laying hens is very important for improving the health of the host and intestine. Mastering the rhythm of fecal microbiota is helpful to strengthen the maintenance of the intestinal health of laying hens, reduce the occurrence of diseases and reduce the use of antibiotics. Generally, our results showed that the relative abundance of a small fraction of the total fecal microbiota oscillated with a rhythm and fewer bacteria oscillated with a 24 h rhythm. Ten replicates per time point were used in our study, which is the same number as the prior mouse study [19,21]. However, one of the main differences between mice and chickens is the applicability of feces samples to the study of gut microbiota. Mouse feces samples apply to human health [59] and are an accepted and reliable source of information about gut microbiota. Together with the applicability of feces samples and the smaller space requirements, it is less challenging to perform more reproducible longitudinal and temporal studies in mouse models than in chicken models. The cecum is the main structure of feces formation, and it plays a vital role in the composition of fecal microbiota. Many studies showed that cecum microbiota was highly like fecal microbiota, and the fecal microbiota is often used to represent intestinal microbiota, especially the composition of cecum microbiota [21,60]. However, the report revealed that fecal microbiotas could not reflect the cecal microbiota in the newly hatched chickens [27]. Therefore, our study could only reflect the situation of the circadian rhythm of fecal microbiota in laying hens. In the future, further study should be conducted to investigate the situation of the cecal microbiota in laying hens.

However, there was no circadian rhythm of microbial community assembly confirmed by JTK_Cycle analysis. A potential explanation for this phenomenon is that the laying hens used in this study were placed on a long-time light program (16L: 8D) and ad libitum feed, whereas light and time-restricted feeding are the two most important factors to regulated the circadian rhythm of host and gut microbiota [61]. To maintain the activity and good production performance of laying hens, the illumination time is usually more than 16 h per day, and sufficient feed is always supplied. If poultry is exposed to light for more than 20 h and free to eat during the period, the activity of the gut microbiota and intestinal function is abnormal, while if they are kept in the dark for at least 6 h every day, the circadian rhythm of poultry will be improved, especially in early life [27]. A previous study reported that time-restrict feeding can change the composition of the gut microbiota and significantly increase the main probiotics, such as *Lactobacillus* and *Bifidobacterium*, but reduce *Helicobacter* [62], in contrast, disrupting the feeding time will cause obesity and metabolic syndrome [63]. Thus, we speculate that feeding food in batches and shortening the illumination time to prolong the dark time and adopting intermittent illumination may be more conducive to a better circadian rhythm of gut microbiota in the future.

## 5. Conclusions

This study examined the circadian rhythm of the fecal microbiota in laying hens, and we systematically characterized the fecal microbiota in 86 samples obtained within 48 h. In our study, there is no obvious circadian rhythm of fecal microbiota in laying hens. The time of defecation may be an important factor that influences the composition and diversity of the feces microbial community. We also systematically proposed the assembly pattern of the microbial community in the oscillations of the fecal microbiota. Abundant ASVs were more shaped based on the dispersal limitation (weak selection), and rare ASVs were the “nod-dominant” fraction. These results might suggest there was no obvious circadian rhythm of fecal microbiota in laying hens under common light and further studies should be conducted to illustrate the reasons and seek the regulated measures to recover the circadian rhythm.

## Data Availability

The sequencing data in this study were deposited in the European Nucleotide Archive (ENA) under accession number PRJEB41206.

## Supplementary Materials

The following are available online at https://www.mdpi.com/article/10.3390/ani11072065/s1. Table S1. Composition and nutrient levels of the diet. Table S2. JTK_Cycle results for the relative abundances of eight predominant bacteria at the phylum level. Table S3. JTK_Cycle results for the top 30 most abundant ASVs. Table S4. JTK_Cycle results for the top 30 most abundant enzymes. Table S5. JTK_Cycle results for the top 30 most important bacterial functions. Figure S1. The rarity curves of 86 samples. Figure S2. The temporal changes of the relative abundance of other six predominant bacteria at the phylum. Figure S3. The temporal changes of the relative abundance of 22 most important ASVs. Figure S4. The temporal changes of the enzymes of contribution. Figure S5. The dominantly related microbiota according to their contribution to these 30 enzymes. Figure S6. The temporal changes of the bacterial functions of contribution.
