# Supplementary material for: Absence of Circadian Rhythm in Fecal Microbiota of Laying Hens under Common Light
Source: Animals (Basel). 2021 Jul 10;11(7):2065. doi: 10.3390/ani11072065 (PMC8300245; doi:10.3390/ani11072065)
Supplement: Supplementary file 1 [file animals-11-02065-s001.zip › animals-1263808-supplementary/Table S1.pdf]

Table S1 Composition and nutrient levels of the diet （air-dry basis）

Ingredients	Composition	Nutrition levels	Nutrition level
Corn（%）	62.00	ME（MJ/kg）	11.30
Soybean meal（%）	26.00	CP（%）	16.20
Limestone（%）	8.00	TP（%）	0.45
Soybean oil（%）	1.00	Ca（%）	3.80
Calcium hydrogen sulfate（%）	1.54	AP（%）	0.32
Methionine（%）	0.16	Met（%）	0.38
Salt（%）	0.30	Lys（%）	0.80
Premix（%）	1.00		
Total（%）	100.00		

Note: The Premix provided the following per kg of the diet: Mn 60 mg，Cu 8 mg, Zn 80 mg, Fe 60 mg, I 0.35 mg, Se 0.3 mg, V_A 9000U, V_{D3} 1600 IU, V_E 5 IU, V_K 0.5 mg, V_{B12} 0.004mg, V_{B6} 3 mg, V_{B2} 25 mg, Biotin 0.1 mg, Folate 0.25 mg, Niacin 20 mg, D-pantothenate 25 mg, Choline chloride 500 mg.
