# Supplementary material for: Absence of Circadian Rhythm in Fecal Microbiota of Laying Hens under Common Light
Source: Animals (Basel). 2021 Jul 10;11(7):2065. doi: 10.3390/ani11072065 (PMC8300245; doi:10.3390/ani11072065)
Supplement: Supplementary file 1 [file animals-11-02065-s001.zip › animals-1263808-supplementary/Table S2.pdf]

Table S2 JTK_cycle results for the relative abundances of eight predominant bacteria at the phylum level

ID	phyla	BH. Q	ADJ.P	PER	LAG	AMP
1	Fusobacteria	0.1105	0.0508	36	21	0.0178
2	Bacteroidetes	0.1380	0.0718	24	3	0.0156
3	Proteobacteria	0.1891	0.1139	30	9	0.0038
4	Euryarchaeota	0.3564	0.2364	24	3	0.0000
5	Actinobacteria	0.7159	0.5333	24	0	0.0040
6	Firmicutes	0.8138	0.6145	36	27	0.0799
7	AD3	1.0000	1.0000	36	9	0.0051
8	Acidobacteria	1.0000	1.0000	36	12	0.0158

Note: BH. Q, Benjamini-Hochberg q value; ADJ.P, Bonferroni-adjusted p value for cyclic oscillations; PER, period length in hours; LAG, lag phase; AMP, amplitude.
