# Supplementary material for: Absence of Circadian Rhythm in Fecal Microbiota of Laying Hens under Common Light
Source: Animals (Basel). 2021 Jul 10;11(7):2065. doi: 10.3390/ani11072065 (PMC8300245; doi:10.3390/ani11072065)
Supplement: Supplementary file 1 [file animals-11-02065-s001.zip › animals-1263808-supplementary/Table S3.pdf]

Table S3 JTK_cycle results for the top 30 most abundant ASVs

ID	ASVs	BH. Q	ADJ.P	PER	LAG	AMP
1	Enterococcus cecorum_6	0.0000	0.0000	24	12	0.0168
2	Enterococcus cecorum_10	0.0003	0.0000	24	12	0.0011
3	Enterococcus_7	0.0063	0.0003	36	0	0.0008
4	Megamonas_24	0.0096	0.0006	36	21	0.0010
5	Burkholderia bryophila_17	0.0144	0.0017	30	0	0.0009
6	Enterobacteriaceae_27	0.0212	0.0032	30	9	0.0009
7	Lactobacillus_9	0.0347	0.0099	36	0	0.0003
8	Lactobacillus_22	0.0518	0.0169	24	3	0.0007
9	Porphyromonadaceae_20	0.0769	0.0267	36	27	0.0001
10	Lachnospiraceae_4	0.0850	0.0303	24	0	0.0022
11	Faecalibacterium_3	0.0850	0.0312	36	21	0.0025
12	Fusobacteriaceae_12	0.0999	0.0396	36	21	0.0070
13	Oscillospira_14	0.0999	0.0402	36	27	0.0003
14	Clostridiales_23	0.1000	0.0449	36	21	0.0005
15	Lactobacillus reuteri_8	0.1148	0.0543	30	27	0.0008
16	Ruminococcaceae_5	0.1380	0.0709	36	21	0.0040
17	Lachnospiraceae_19	0.1418	0.0767	36	21	0.0019
18	Planococcaceae_26	0.1428	0.0787	0	0	0.0000
19	Clostridium_21	0.1515	0.0850	36	21	0.0037
20	Ruminococcus gnavus_2	0.1696	0.1004	36	21	0.0003
21	Clostridium colinum_13	0.2149	0.1338	36	21	0.0002
22	Bacteroidales S247	0.2167	0.1371	30	3	0.0001
23	Bradyrhizobiaceae_15	0.3831	0.2590	30	6	0.0004
24	Lactobacillus_29	0.3949	0.2740	24	3	0.0008
25	Salinispora tropica_25	0.4878	0.3534	0	0	0.0000
26	AD3 ABS6	1.0000	0.8007	24	15	0.0001
27	Clostridiaceae SMB53_11	1.0000	0.9230	0	0	0.0000
28	Sutterella_1	1.0000	1.0000	36	15	0.0007
29	Nitrospiraceae JG37-AG-70	1.0000	1.0000	30	6	0.0007
30	Enterobacteriaceae_28	1.0000	1.0000	36	6	0.0004

Note: BH. Q, Benjamini-Hochberg q value; ADJ.P, Bonferroni-adjusted p value for cyclic oscillations; PER, period length in hours; LAG, lag phase; AMP, amplitude.
