# Supplementary material for: Absence of Circadian Rhythm in Fecal Microbiota of Laying Hens under Common Light
Source: Animals (Basel). 2021 Jul 10;11(7):2065. doi: 10.3390/ani11072065 (PMC8300245; doi:10.3390/ani11072065)
Supplement: Supplementary file 1 [file animals-11-02065-s001.zip › animals-1263808-supplementary/Table S4.pdf]

Table S4 JTK_cycle results for the top 30 most abundant enzymes

ID	ec annotation	BH. Q	ADJ.P	PER	LAG	AMP
1	Ureidoglycolate dehydrogenase (NAD(+))	0.0096	0.0007	30	9	59.1212
2	Methylmalonyl-CoA carboxytransferase	0.0140	0.0014	36	21	79.0687
3	Lipopolysaccharide 3-alpha-galactosyltransferase	0.0140	0.0014	36	21	157.4727
4	N(6)-hydroxylysine O-acetyltransferase	0.0212	0.0035	30	9	20.5061
5	L-ribulose-5-phosphate 3-epimerase	0.0260	0.0054	30	9	29.2601
6	Sulfofructosephosphate aldolase	0.0260	0.0059	30	9	20.1243
7	Arabinonate dehydratase	0.0260	0.0064	30	24	149.9066
8	Anhydro-N-acetylmuramic acid kinase	0.0260	0.0064	24	15	1475.3553
9	Hydroxydechloroatrazine ethylaminohydrolase	0.0272	0.0070	30	24	150.7340
10	Fumarate reductase (CoM/CoB)	0.0448	0.0137	24	3	80.2566
11	Sulfolactaldehyde 3-reductase	0.0448	0.0137	30	9	18.3848
12	L-xylulokinase	0.0482	0.0152	36	3	100.5082
13	Caffeoyl-CoA O-methyltransferase	0.0966	0.0365	36	21	1332.2033
14	2.7.11.30 and 2.7.12.1	0.0999	0.0408	36	21	1353.1195
15	Methylaspartate ammonia-lyase	0.1000	0.0431	36	21	1406.7889
16	Carbon-monoxide dehydrogenase (acceptor)	0.1000	0.0431	36	21	10951.7123
17	D-glucosaminate-6-phosphate ammonia lyase	0.1000	0.0443	36	21	1364.7161
18	Phospholipase D	0.1148	0.0550	30	0	73.3270
19	Methylaspartate mutase	0.1177	0.0581	36	21	2743.3622
20	Urocanate reductase	0.1389	0.0737	36	15	2134.7554
21	16S rRNA (cytosine(1407)-C(5))-methyltransferase	0.1599	0.0930	30	9	29.3308
22	Membrane dipeptidase	0.2004	0.1227	36	21	5390.5932
23	UDP-galactopyranose mutase	0.2704	0.1766	30	27	5945.4599
24	Homocitrate synthase	0.3831	0.2619	36	18	520.6639
25	tRNA(adenine(34)) deaminase	0.5188	0.3811	36	24	5806.1033
26	Non-specific protein-tyrosine kinase	1.0000	0.8479	24	15	0.5303
27	N-carbamoylsarcosine amidase	1.0000	1.0000	36	9	14.8492
28	Aliphatic aldoxime dehydratase	1.0000	1.0000	36	6	31.1127
29	Glucosylglycerol-phosphate synthase	1.0000	1.0000	36	15	13.4350
30	Sterol 3-beta-glucosyltransferase	1.0000	1.0000	36	9	47.3762

Note: BH. Q, Benjamini-Hochberg q value; ADJ.P, Bonferroni-adjusted p value for cyclic oscillations; PER, period length in hours; LAG, lag phase; AMP, amplitude.
