# Supplementary material for: Absence of Circadian Rhythm in Fecal Microbiota of Laying Hens under Common Light
Source: Animals (Basel). 2021 Jul 10;11(7):2065. doi: 10.3390/ani11072065 (PMC8300245; doi:10.3390/ani11072065)
Supplement: Supplementary file 1 [file animals-11-02065-s001.zip › animals-1263808-supplementary/Table S5 .pdf]

Table S5 JTK_cycle results for the top 30 most important bacterial functions

ID	pathway annotation	BH. Q	ADJ.P	PER	LAG	AMP
1	cob(II)yrinate a,c-diamide biosynthesis I (early cobalt insertion)	0.0063	0.0002	36	21	7478.3619
2	allantoin degradation IV (anaerobic)	0.0096	0.0005	30	9	64.8903
3	glutaryl-CoA degradation	0.0126	0.0010	36	18	2522.1568
4	L-glutamate degradation V (via hydroxyglutarate)	0.0144	0.0018	36	21	3870.1754
5	enterobacterial common antigen biosynthesis	0.0182	0.0024	36	3	143.5266
6	aerobactin biosynthesis	0.0212	0.0033	30	9	21.3841
7	superpathway of L-arginine and L-ornithine degradation	0.0250	0.0046	36	3	190.5663
8	superpathway of L-arginine, putrescine, and 4-aminobutanoate degradation	0.0250	0.0046	36	3	190.5663
9	L-glutamate degradation VIII (to propanoate)	0.0253	0.0049	36	21	338.9865
10	polymyxin resistance	0.0260	0.0064	36	3	149.8873
11	L-lysine fermentation to acetate and butanoate	0.0272	0.0072	36	21	3544.2093
12	superpathway of L-tryptophan biosynthesis	0.0280	0.0077	30	9	240.4299
13	sulfoglycolysis	0.0610	0.0205	30	9	10.9508
14	phospholipases	0.1177	0.0589	30	0	168.3977
15	adenosine nucleotides degradation IV	0.1599	0.0930	36	21	111.0606
16	ppGpp biosynthesis	0.2704	0.1766	24	15	2795.2599
17	superpathway of (Kdo)2-lipid A biosynthesis	0.4351	0.3064	30	9	154.4392
18	polyisoprenoid biosynthesis (E. coli)	0.4385	0.3132	36	27	8087.3841
19	superpathway of polyamine biosynthesis II	0.8192	0.6269	36	21	1102.4154
20	superpathway of hexuronide and hexuronate degradation	0.8581	0.6655	36	3	1463.5915
21	superpathway of pyrimidine ribonucleotides de novo biosynthesis	0.9526	0.7484	36	18	1321.7372
22	reductive acetyl coenzyme A pathway	1.0000	0.9230	36	12	787.0604
23	creatinine degradation II	1.0000	1.0000	36	9	43.8521
24	methylaspartate cycle	1.0000	1.0000	36	15	999.3760
25	superpathway of sulfolactate degradation	1.0000	1.0000	30	3	129.4658
26	D-arabinose degradation III	1.0000	1.0000	36	15	35.8433
27	superpathway of taurine degradation	1.0000	1.0000	36	9	86.6653
28	purine nucleobases degradation I (anaerobic)	1.0000	1.0000	24	0	3782.1198
29	nylon-6 oligomer degradation	1.0000	1.0000	36	9	143.1437
30	ethylmalonyl-CoA pathway	1.0000	1.0000	30	3	543.8440

Note: BH. Q, Benjamini-Hochberg q value; ADJ.P, Bonferroni-adjusted p value for cyclic oscillations; PER, period length in hours; LAG, lag phase; AMP, amplitude.
