# Supplementary figures and images for: Absence of Circadian Rhythm in Fecal Microbiota of Laying Hens under Common Light
Source: Animals (Basel). 2021 Jul 10;11(7):2065. doi: 10.3390/ani11072065 (PMC8300245; doi:10.3390/ani11072065)

Bacteroidetes

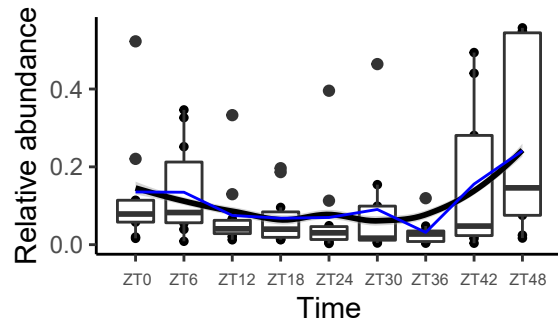

Fusobacteria

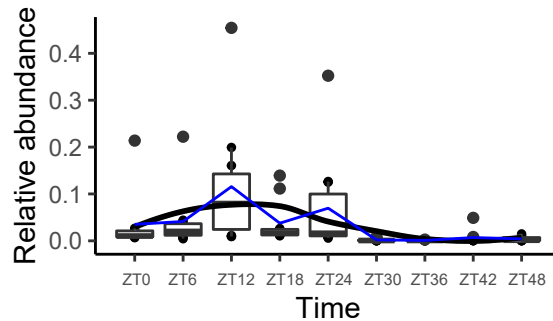

Acidobacteria

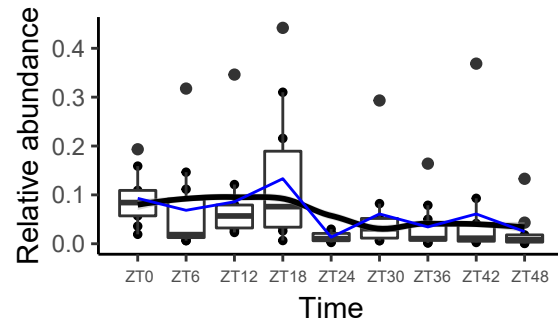

AD3

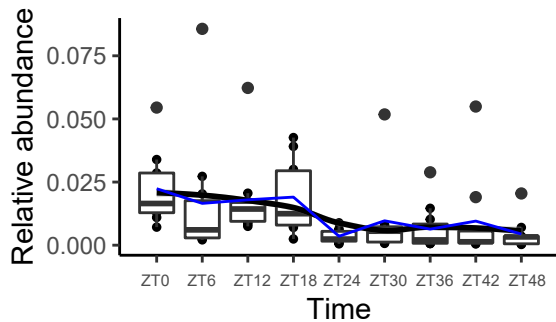

Actinobacteria

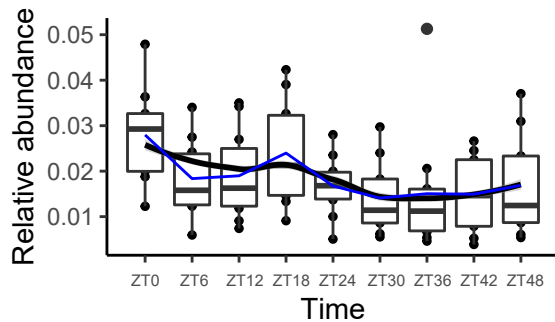

Euryarchaeota

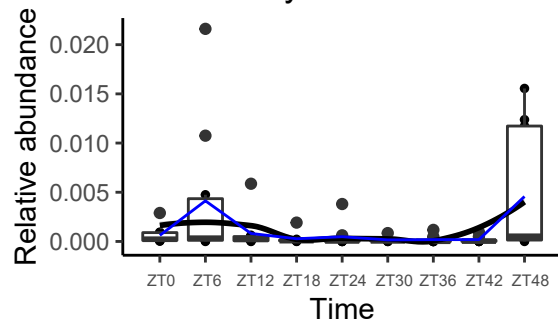

Supplement: Supplementary file 1 [file animals-11-02065-s001.zip › animals-1263808-supplementary/Figure S1.pdf]

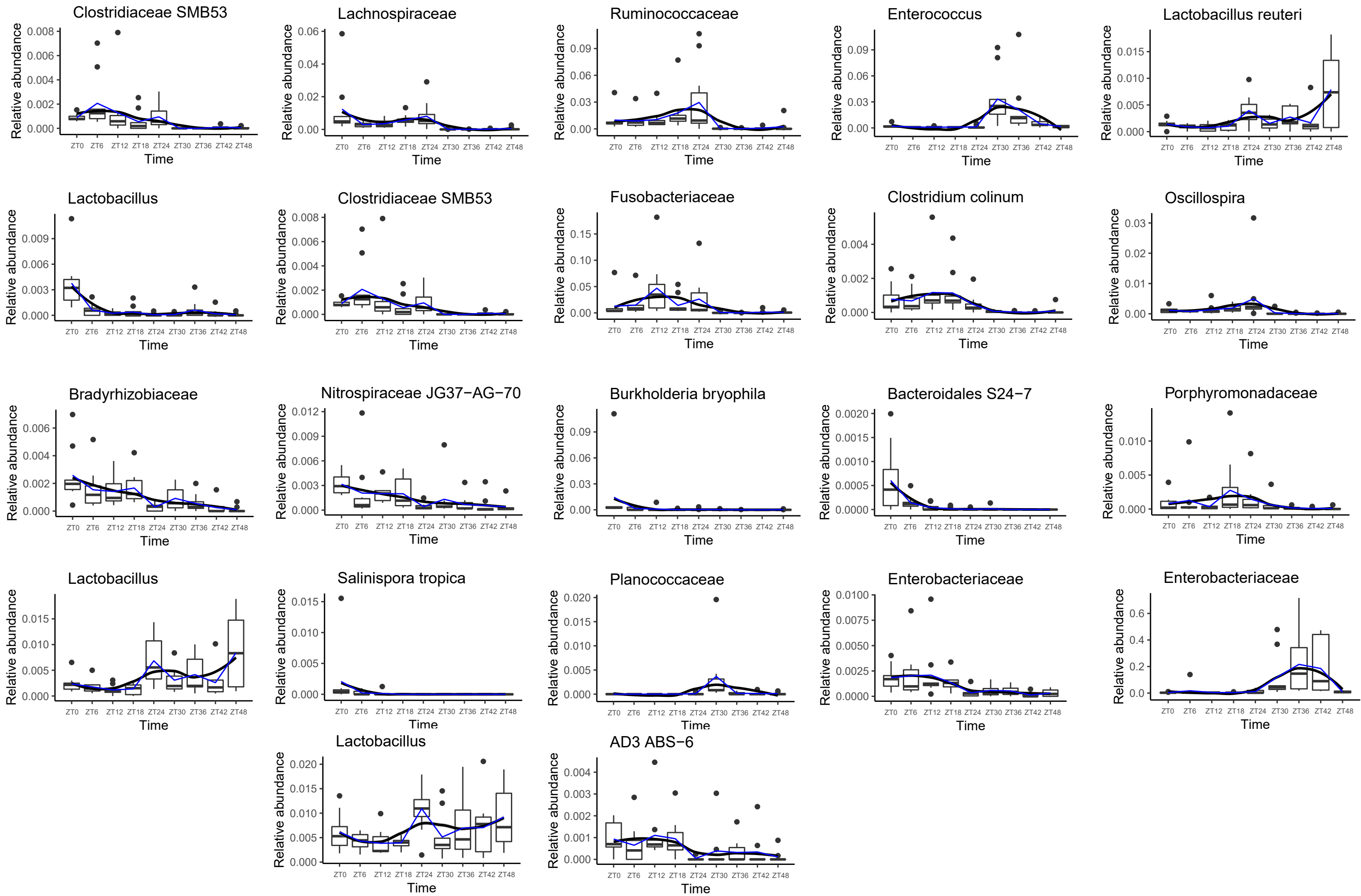

Supplement: Supplementary file 1 [file animals-11-02065-s001.zip › animals-1263808-supplementary/Figure S2.pdf]

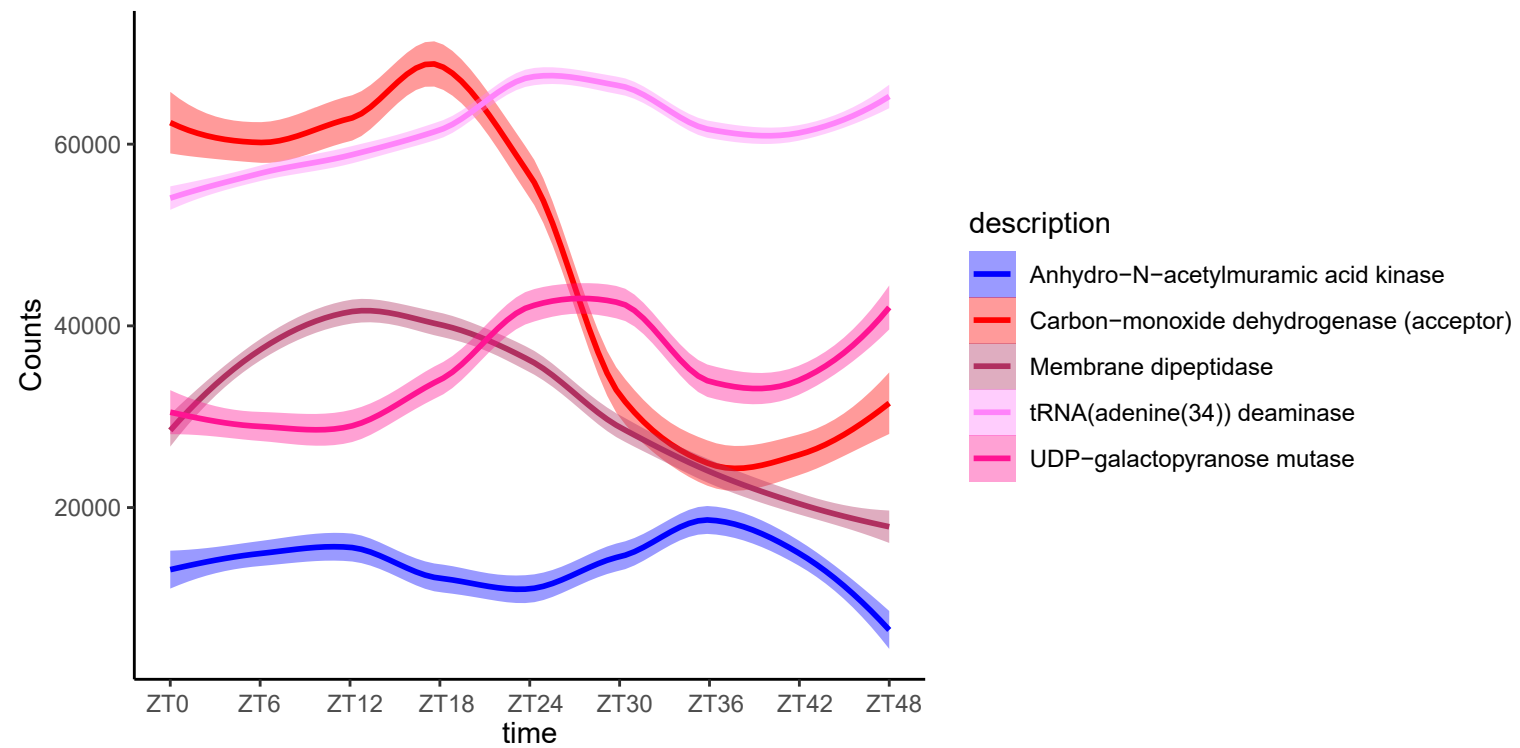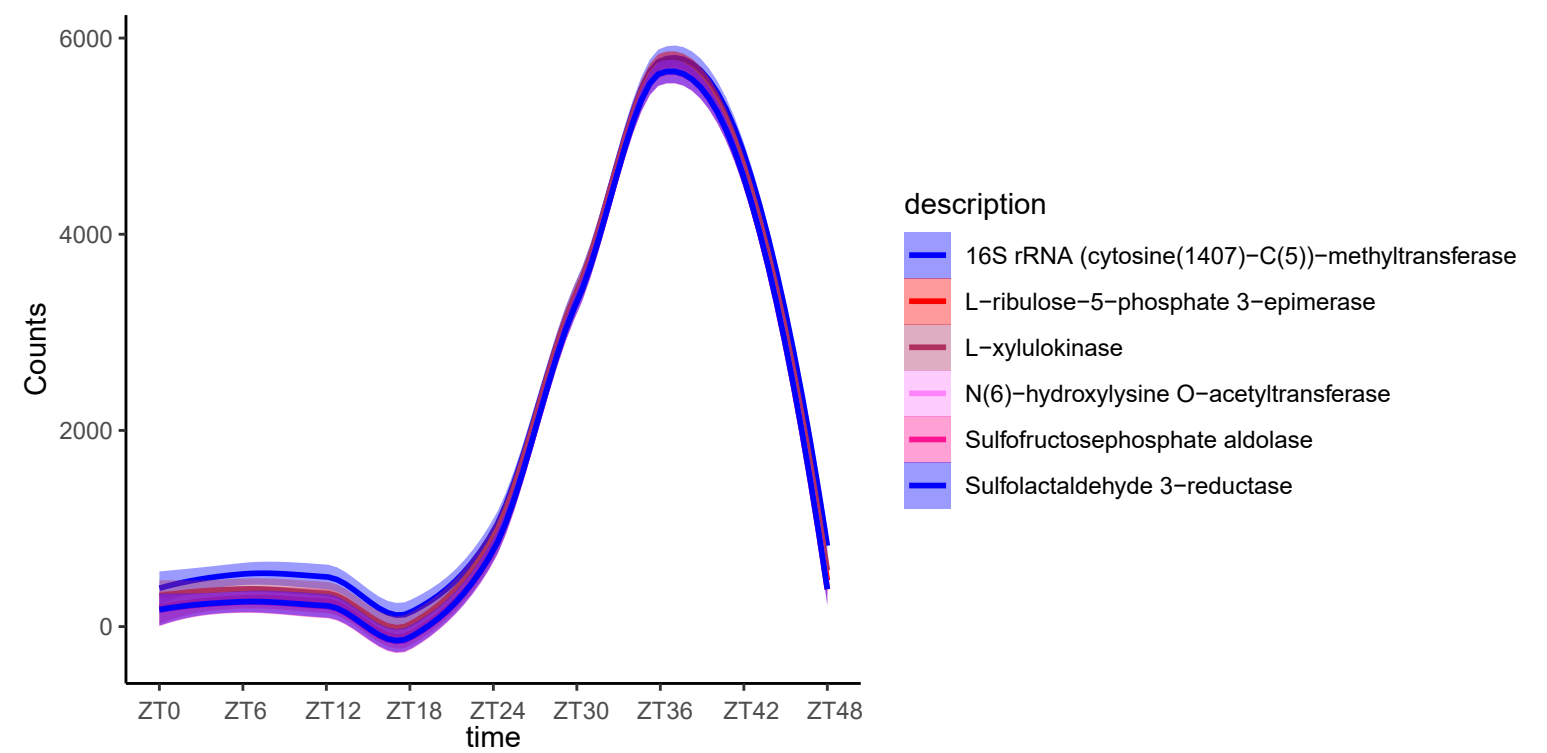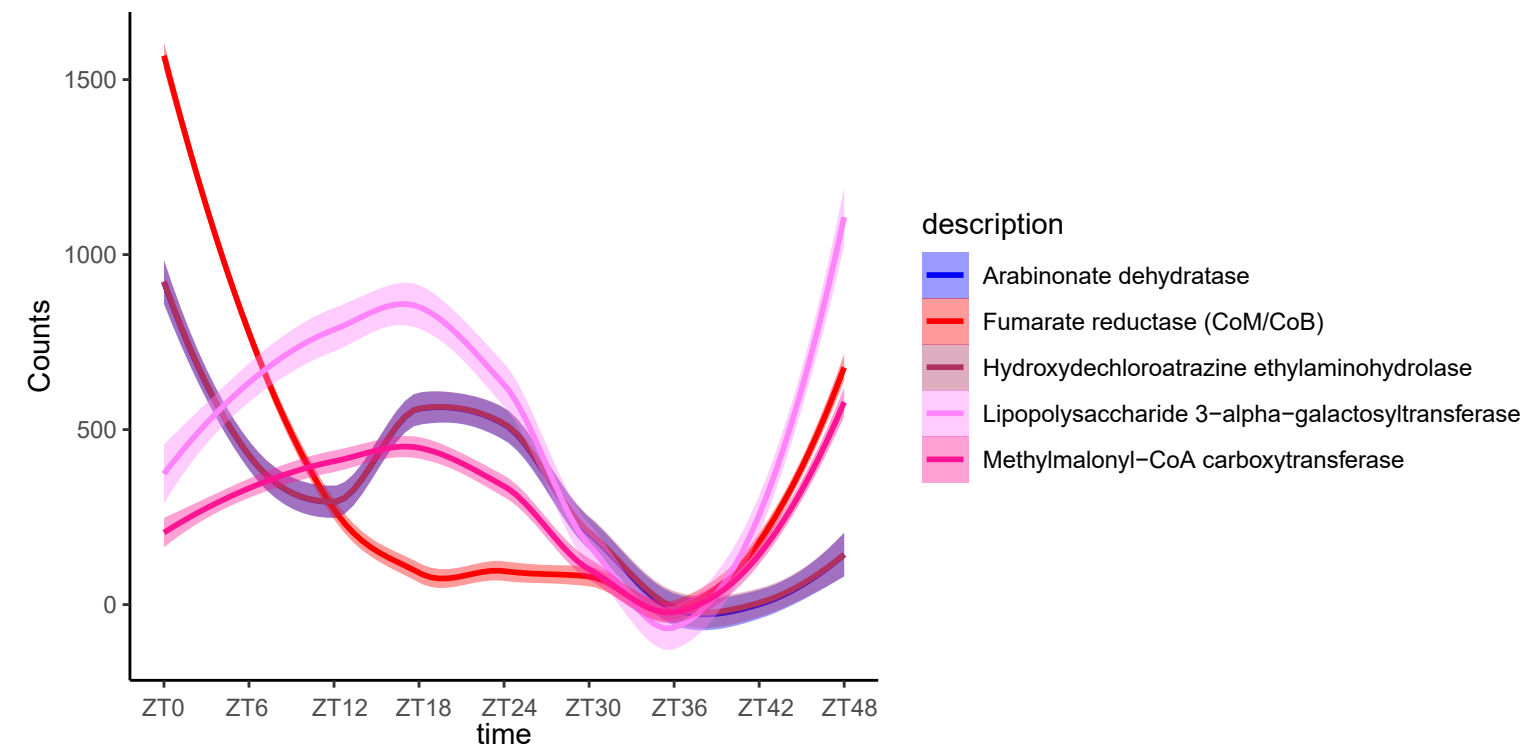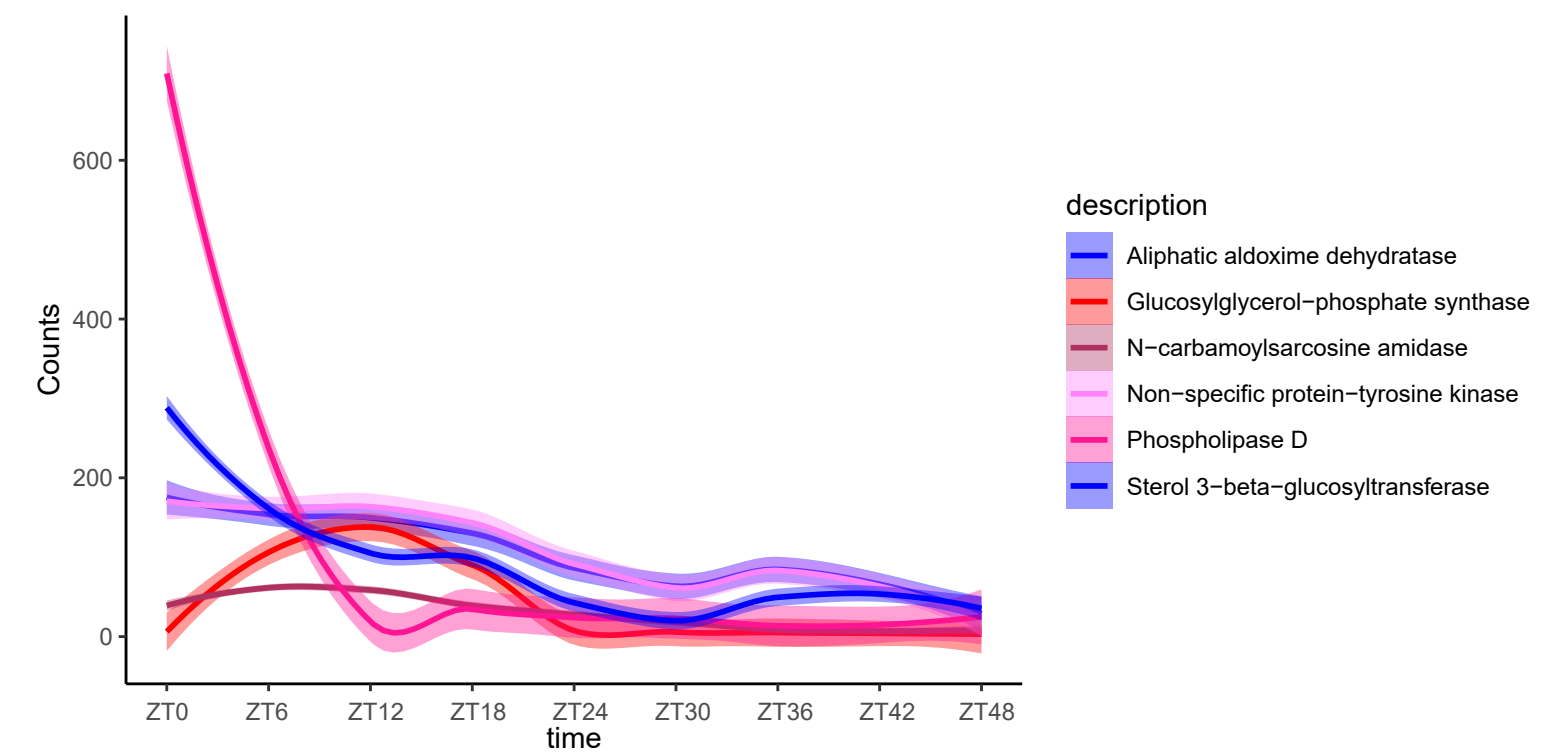

Supplement: Supplementary file 1 [file animals-11-02065-s001.zip › animals-1263808-supplementary/Figure S3.pdf]

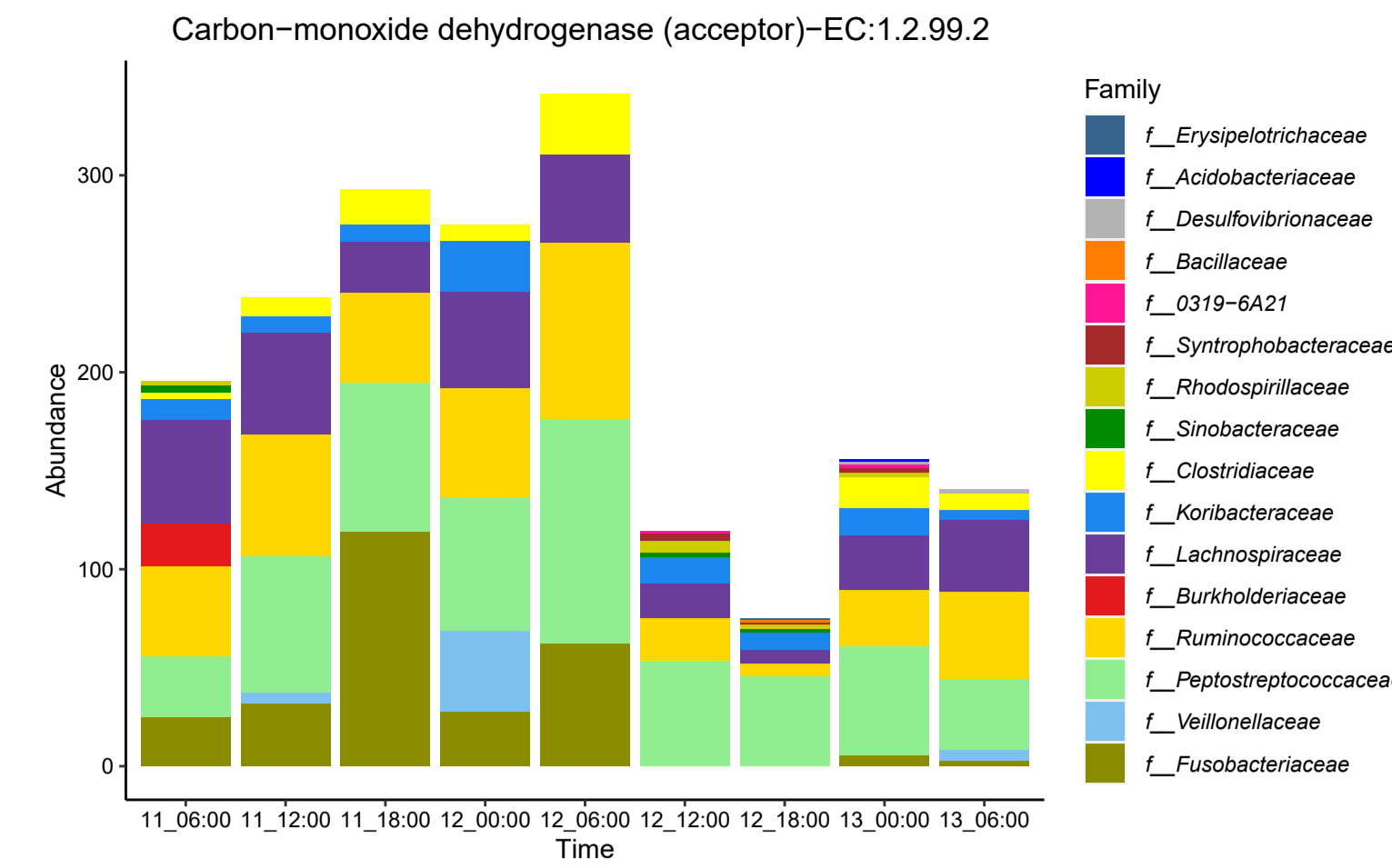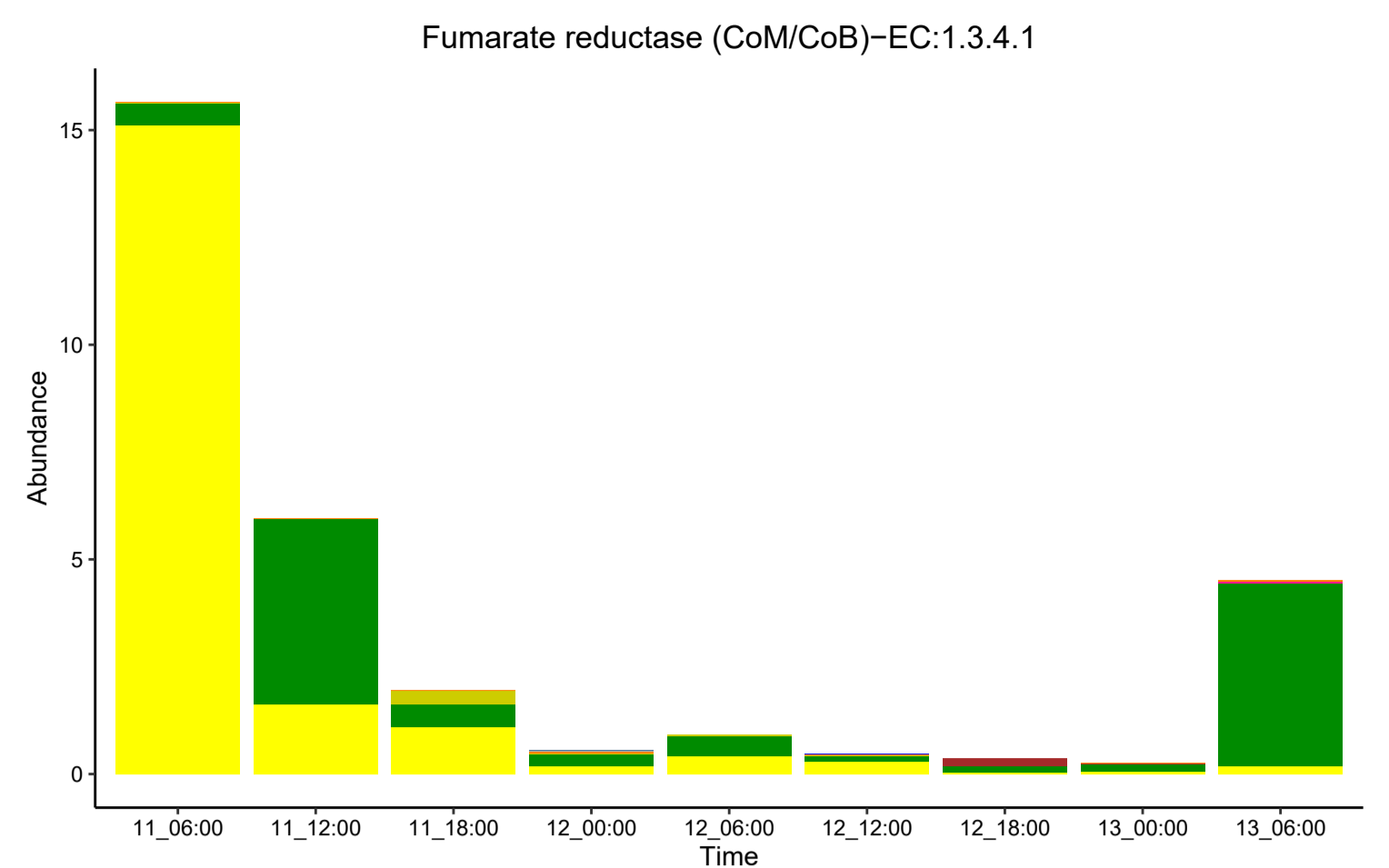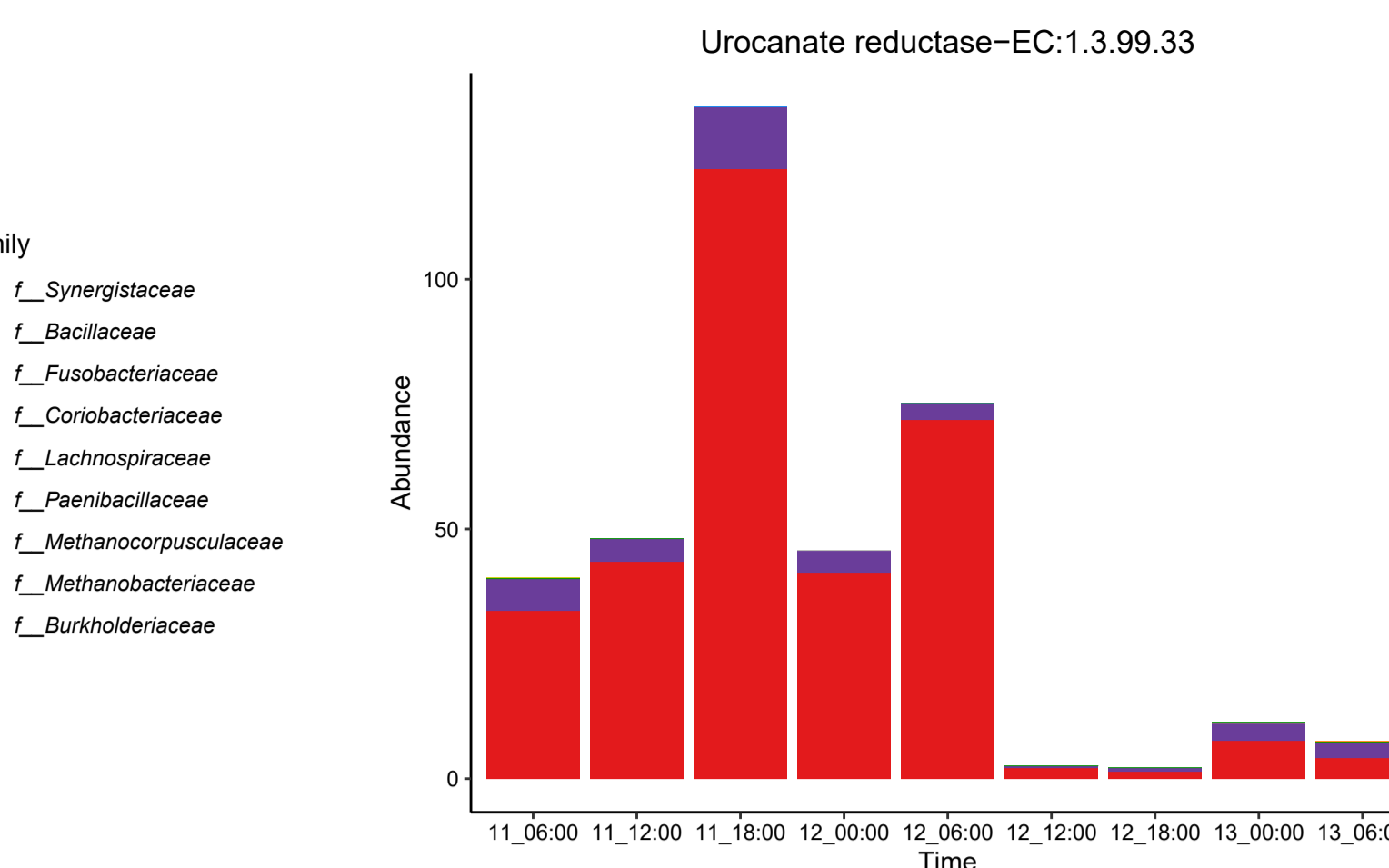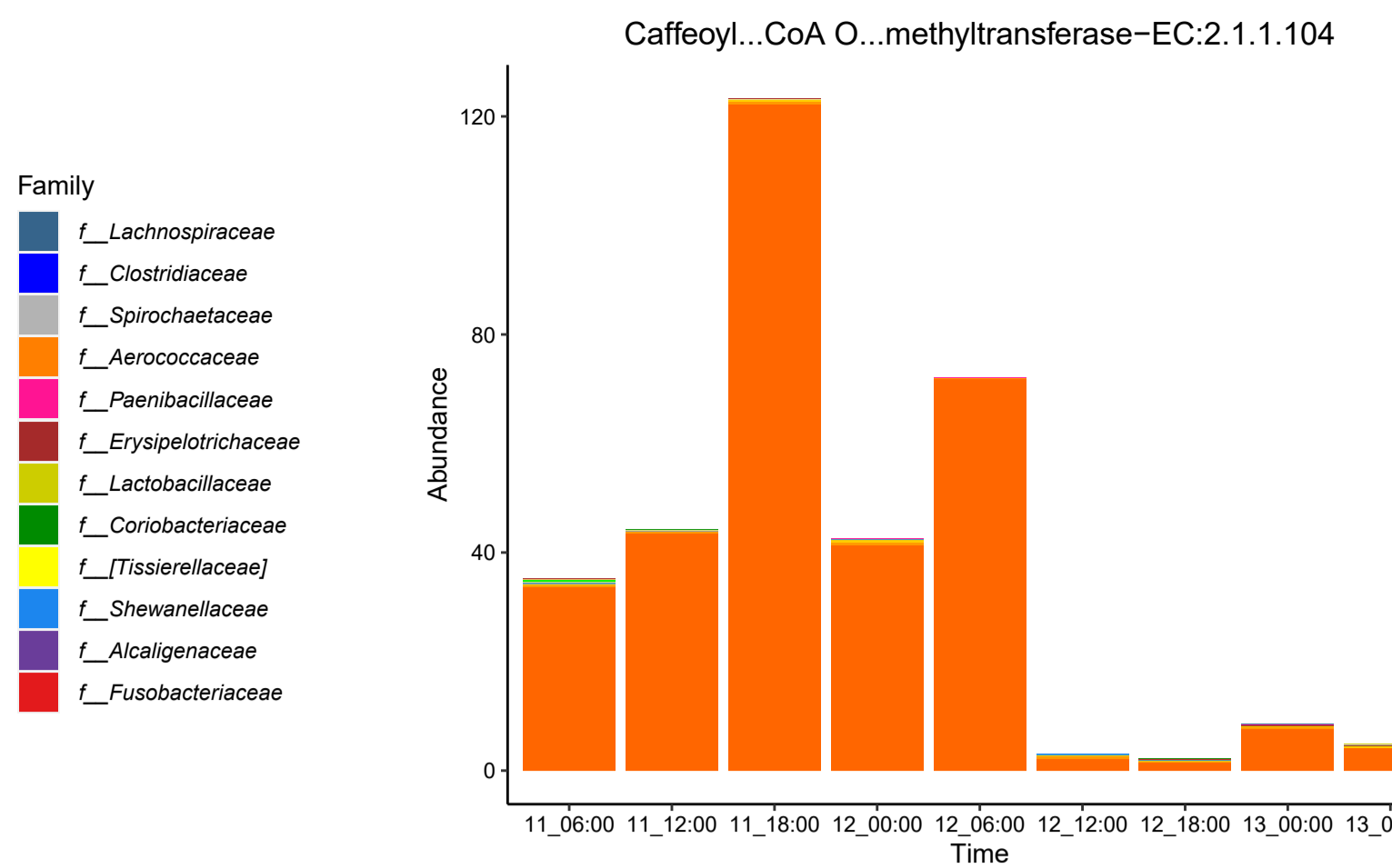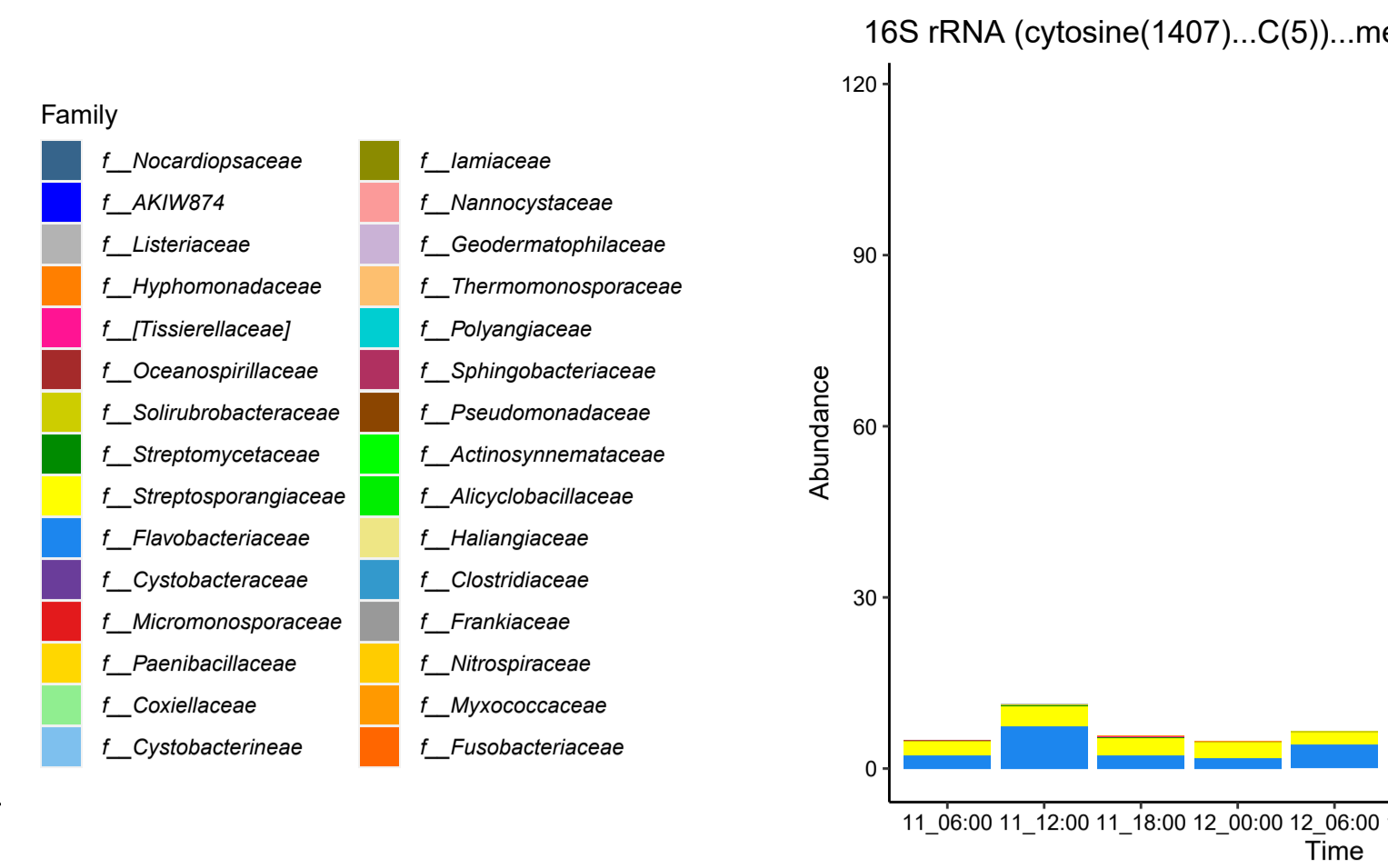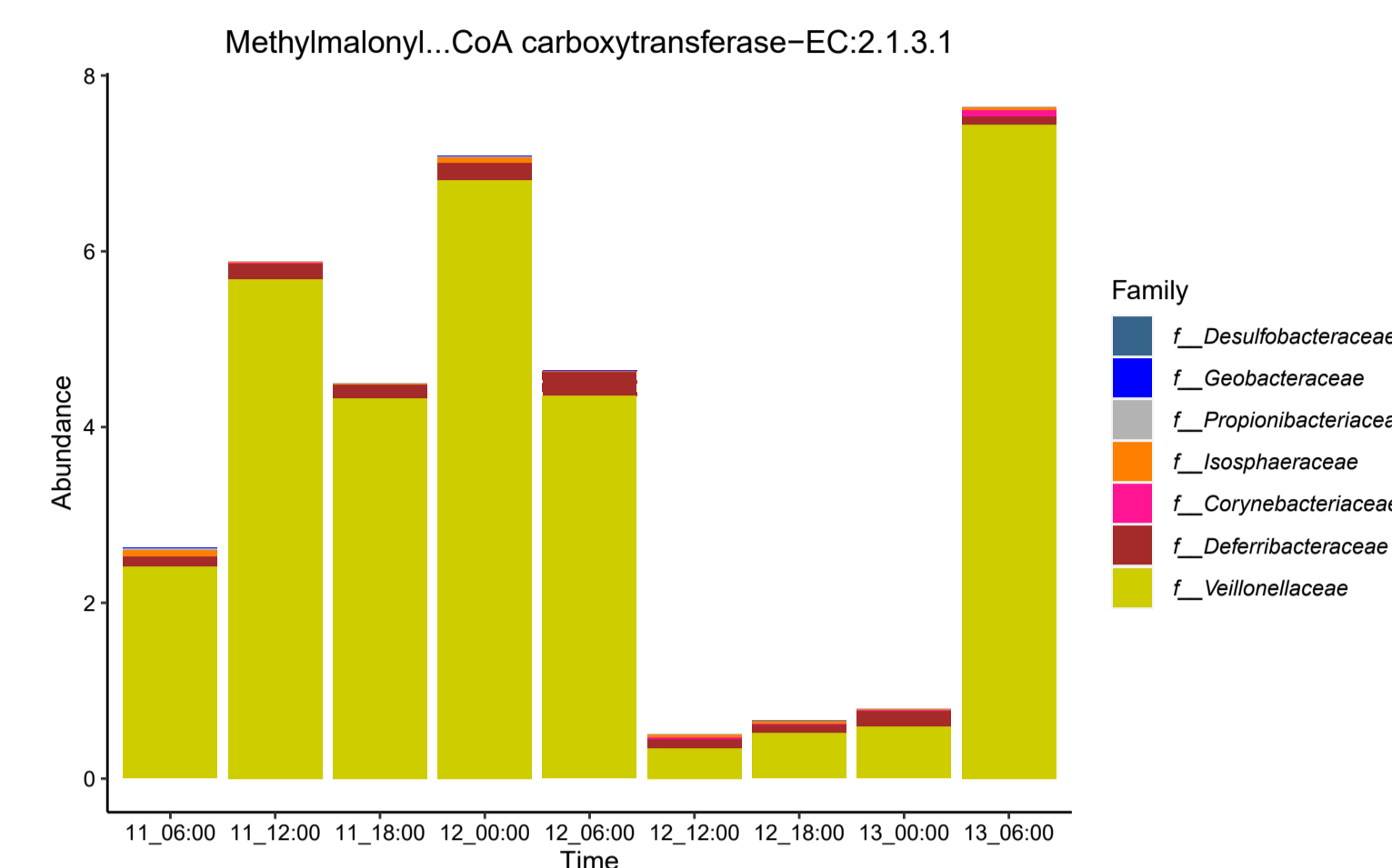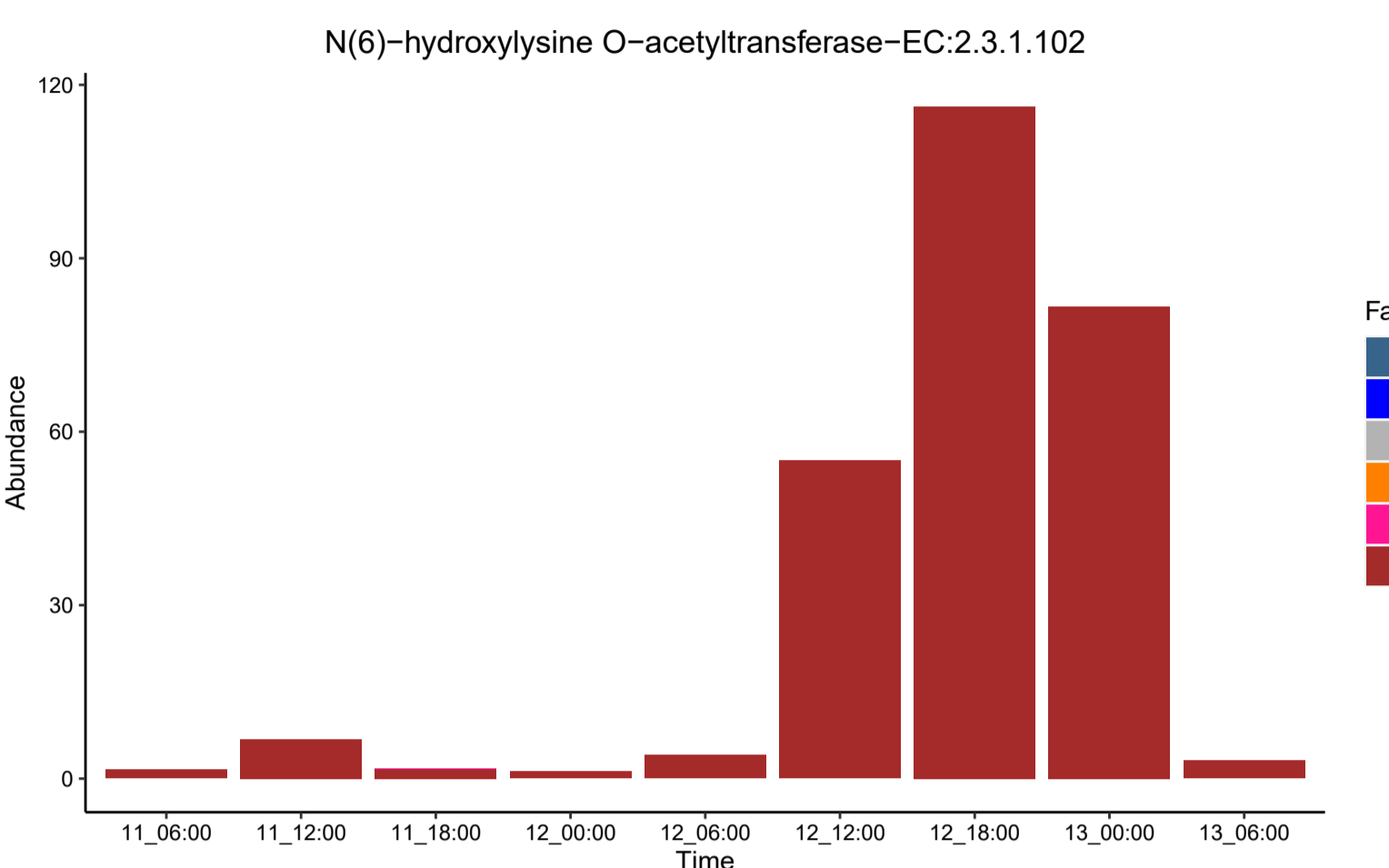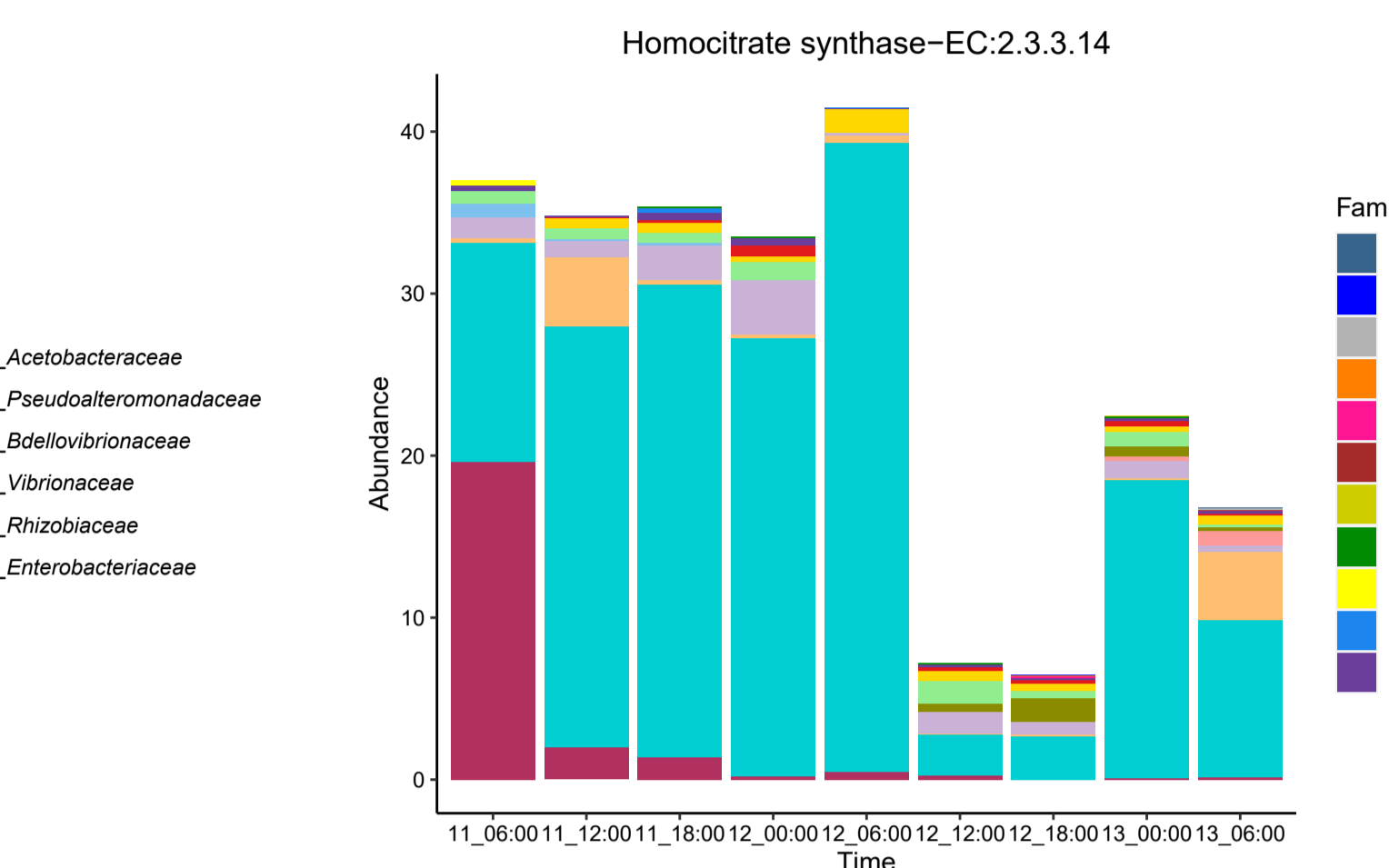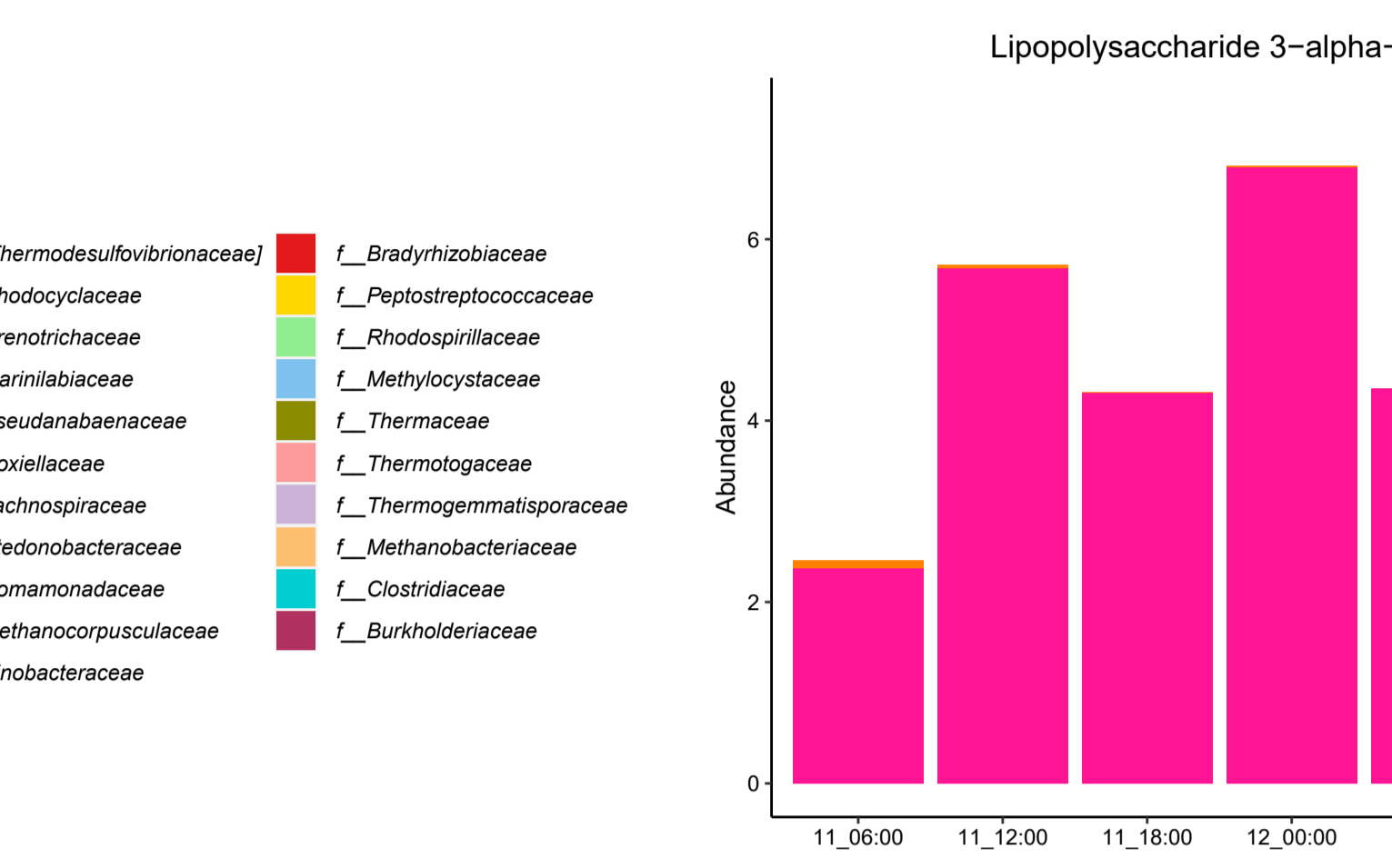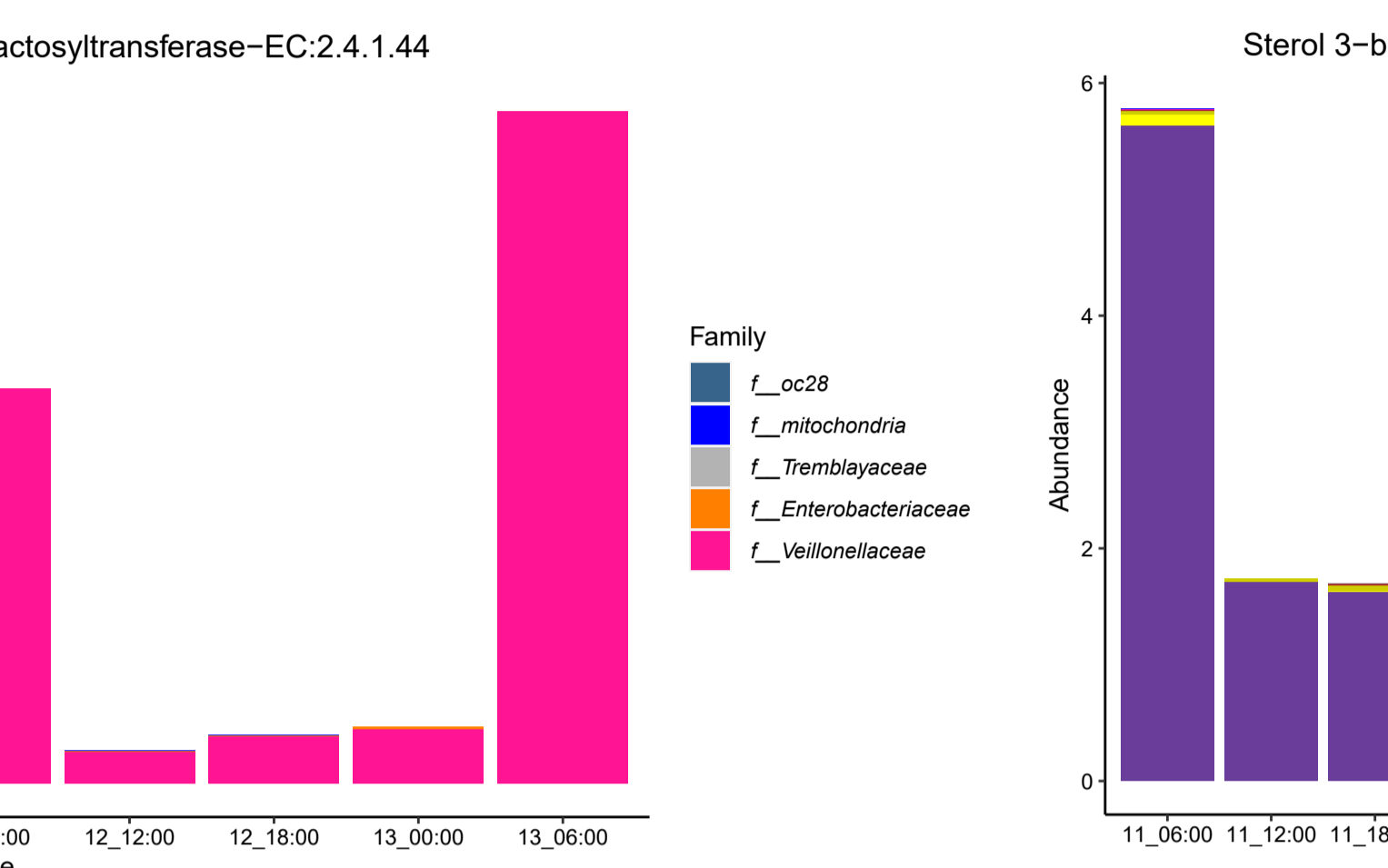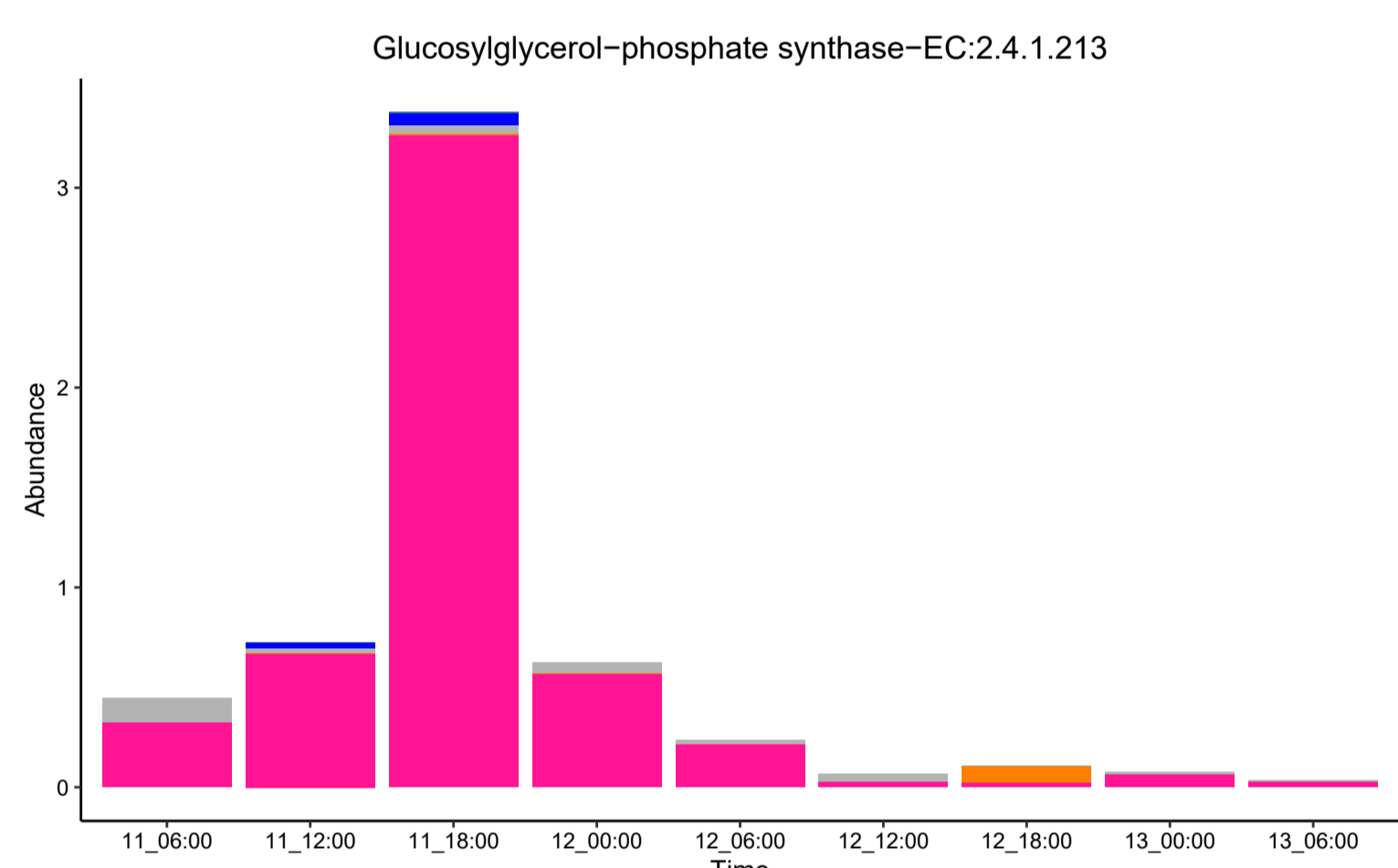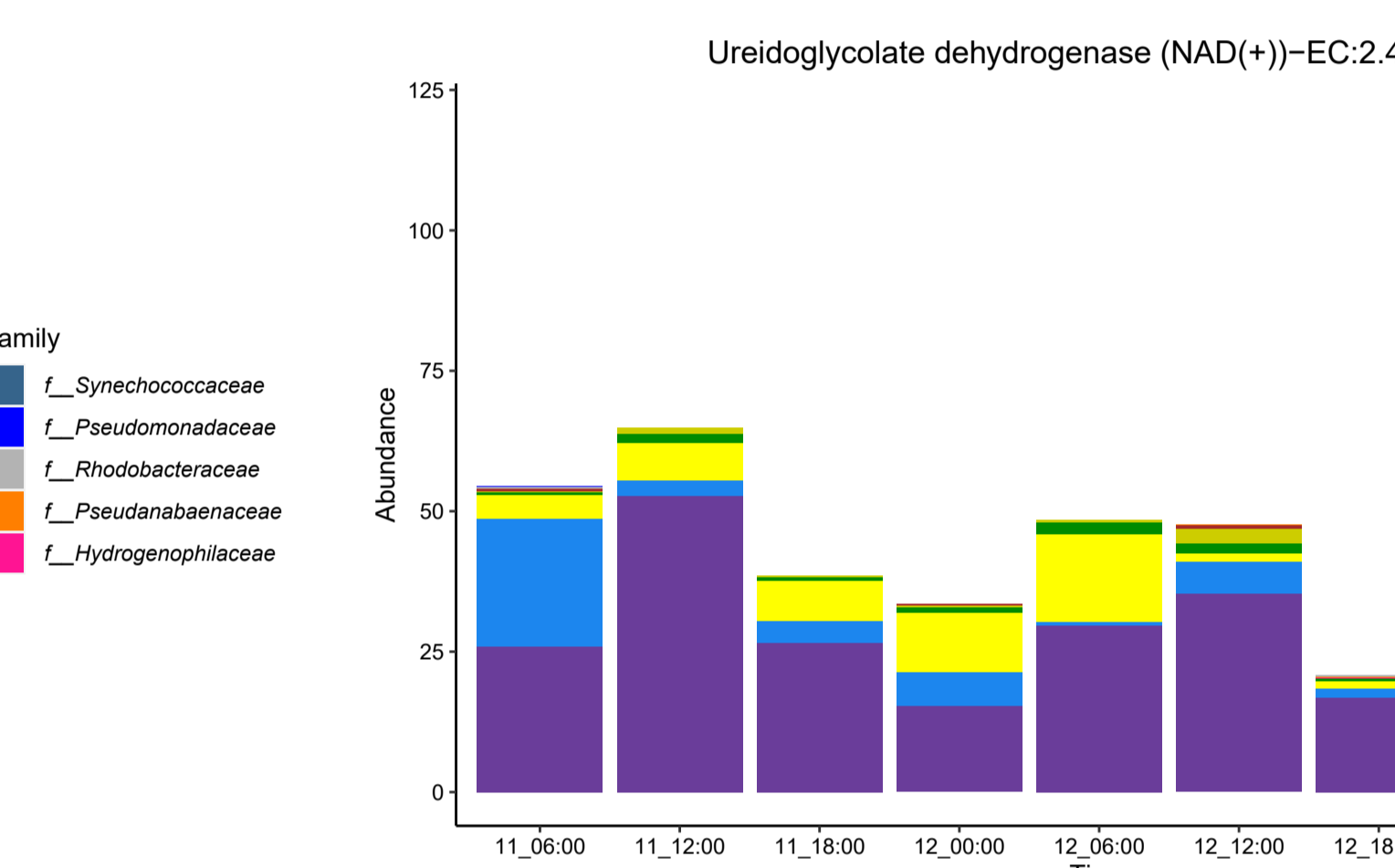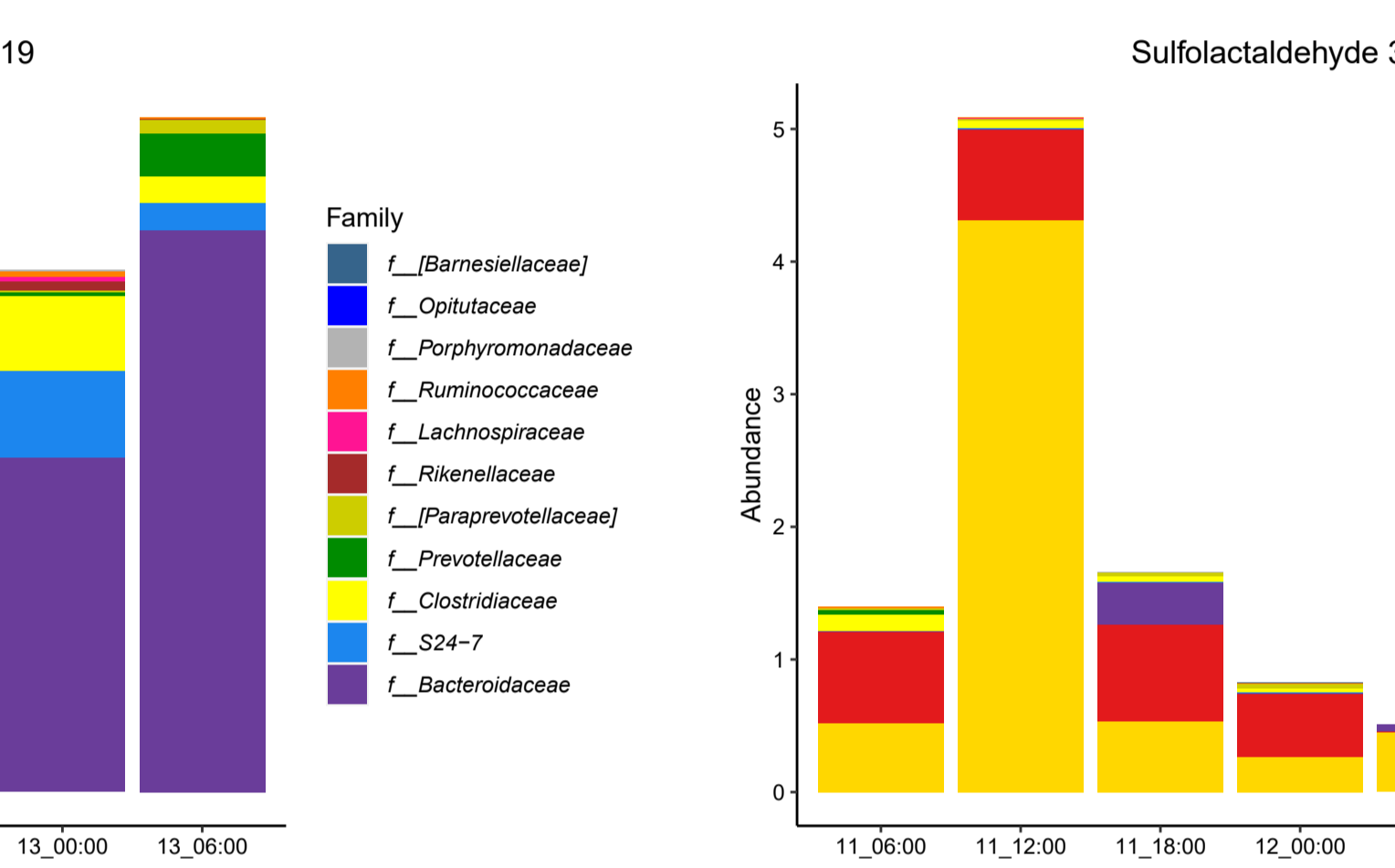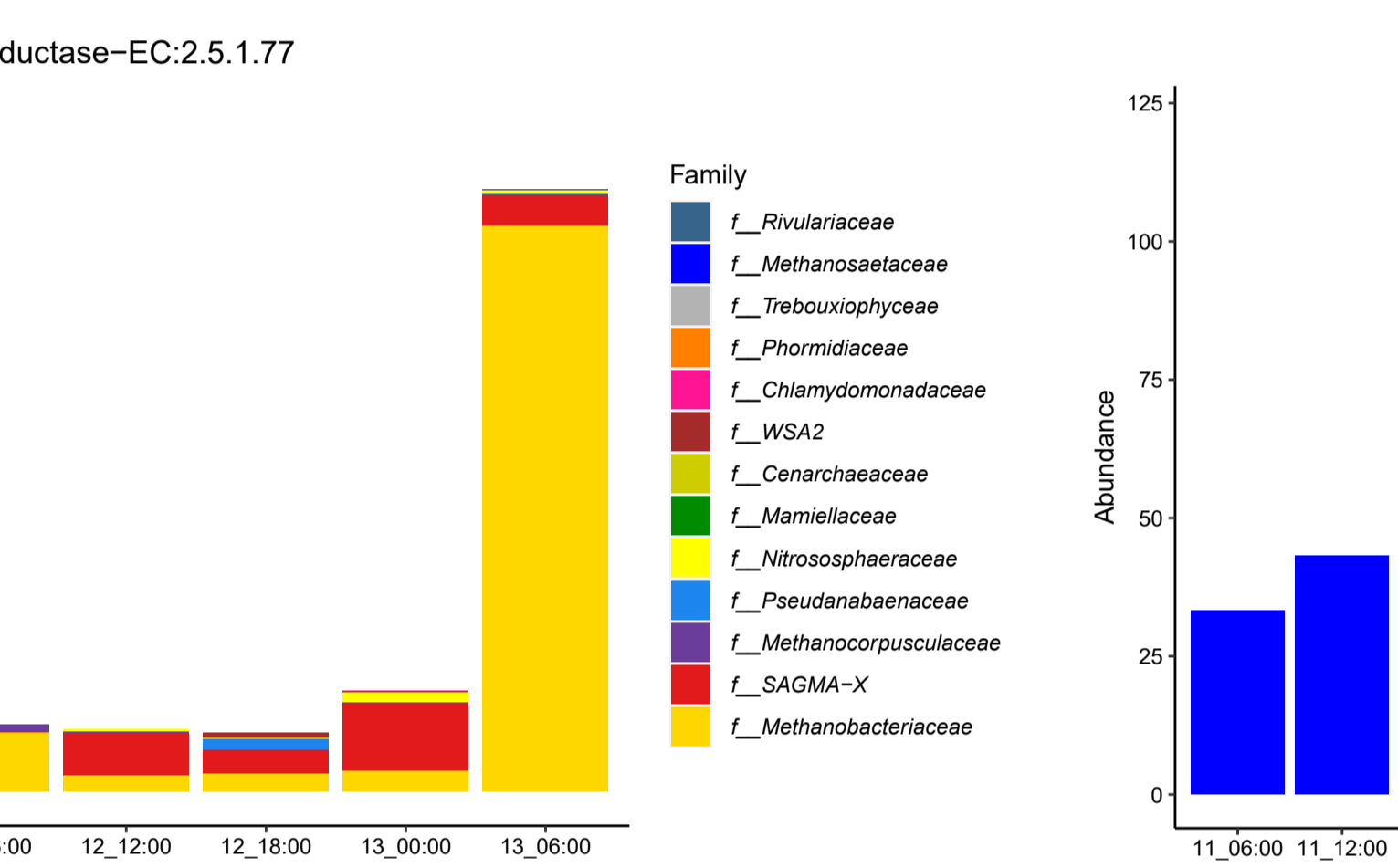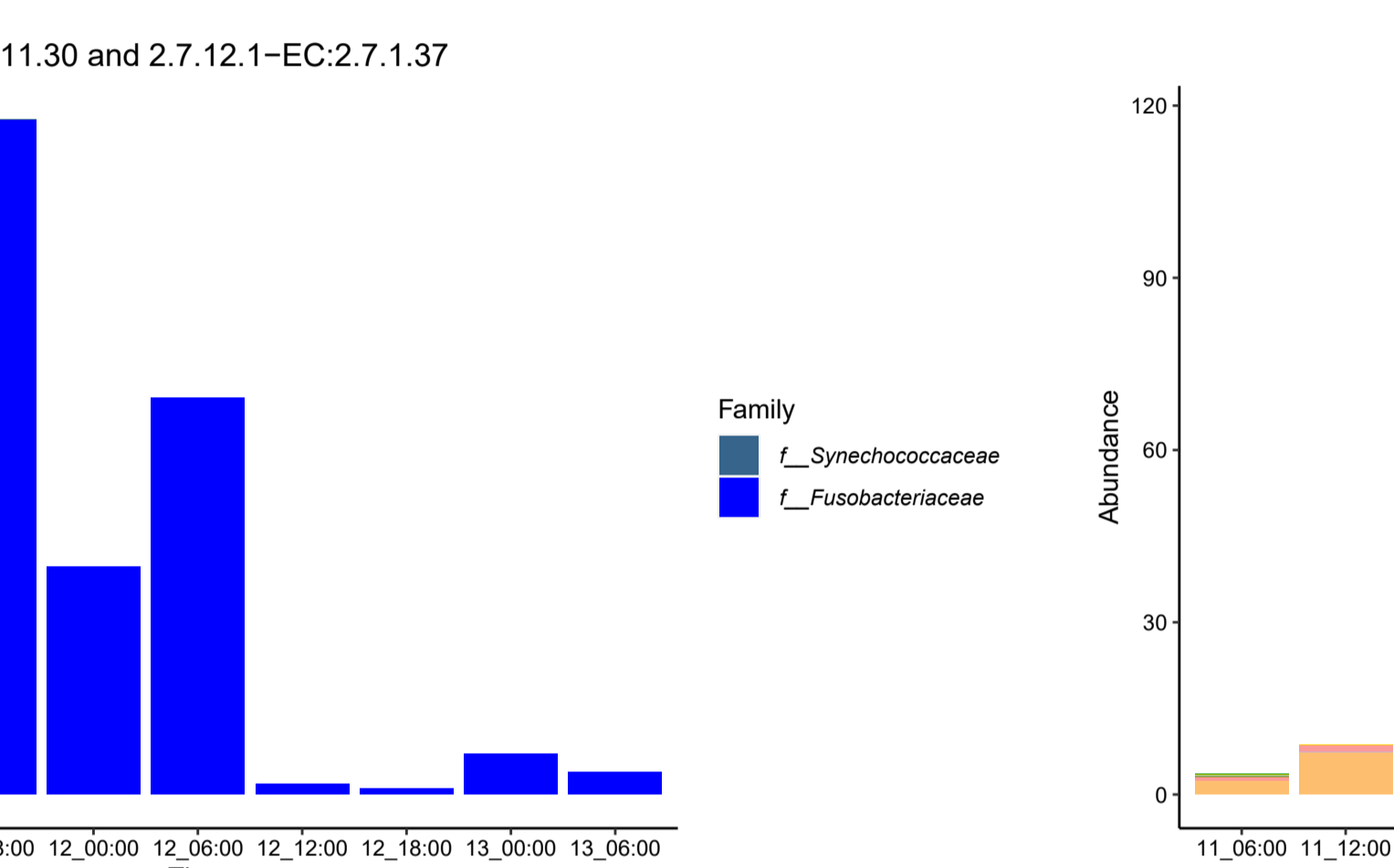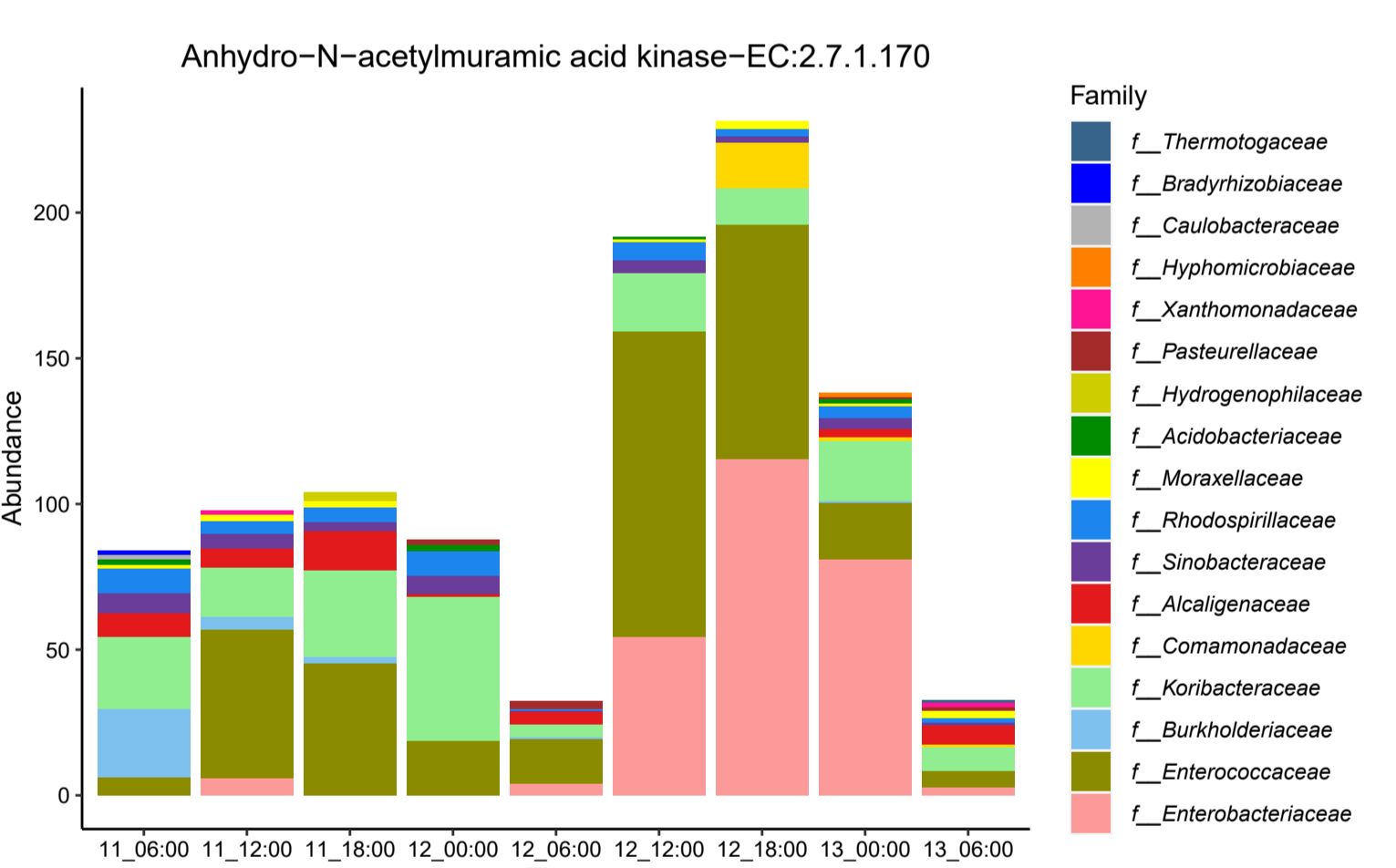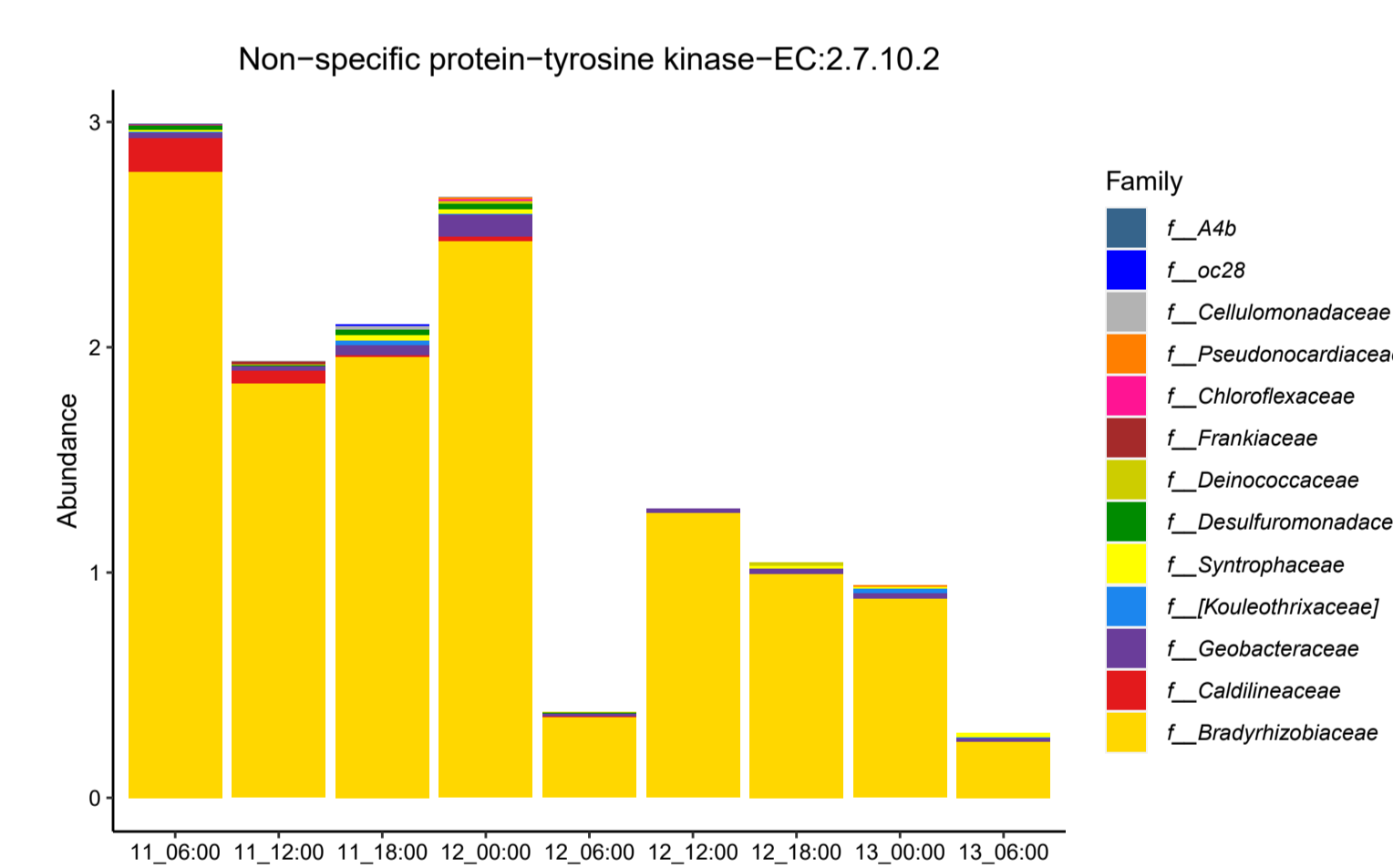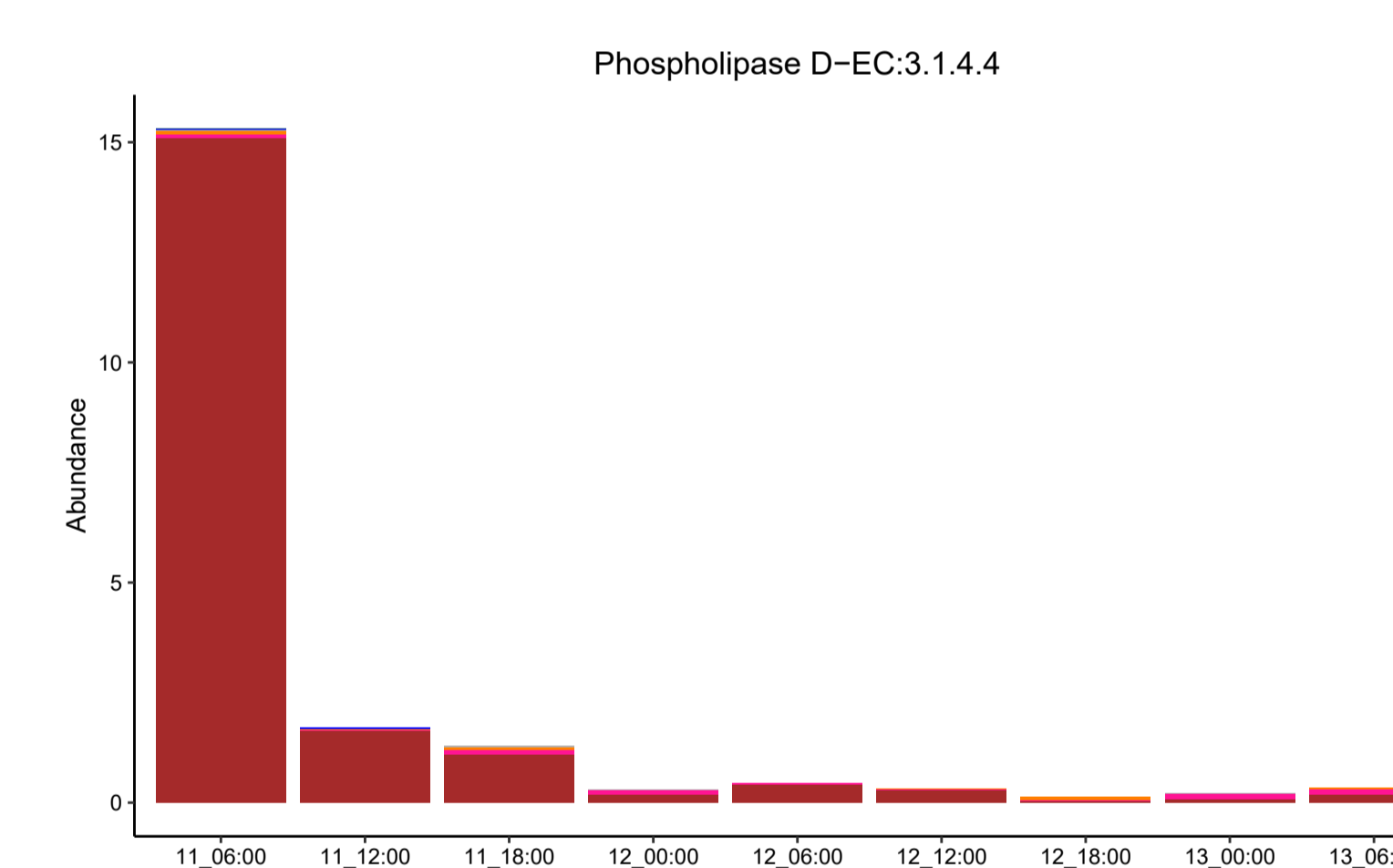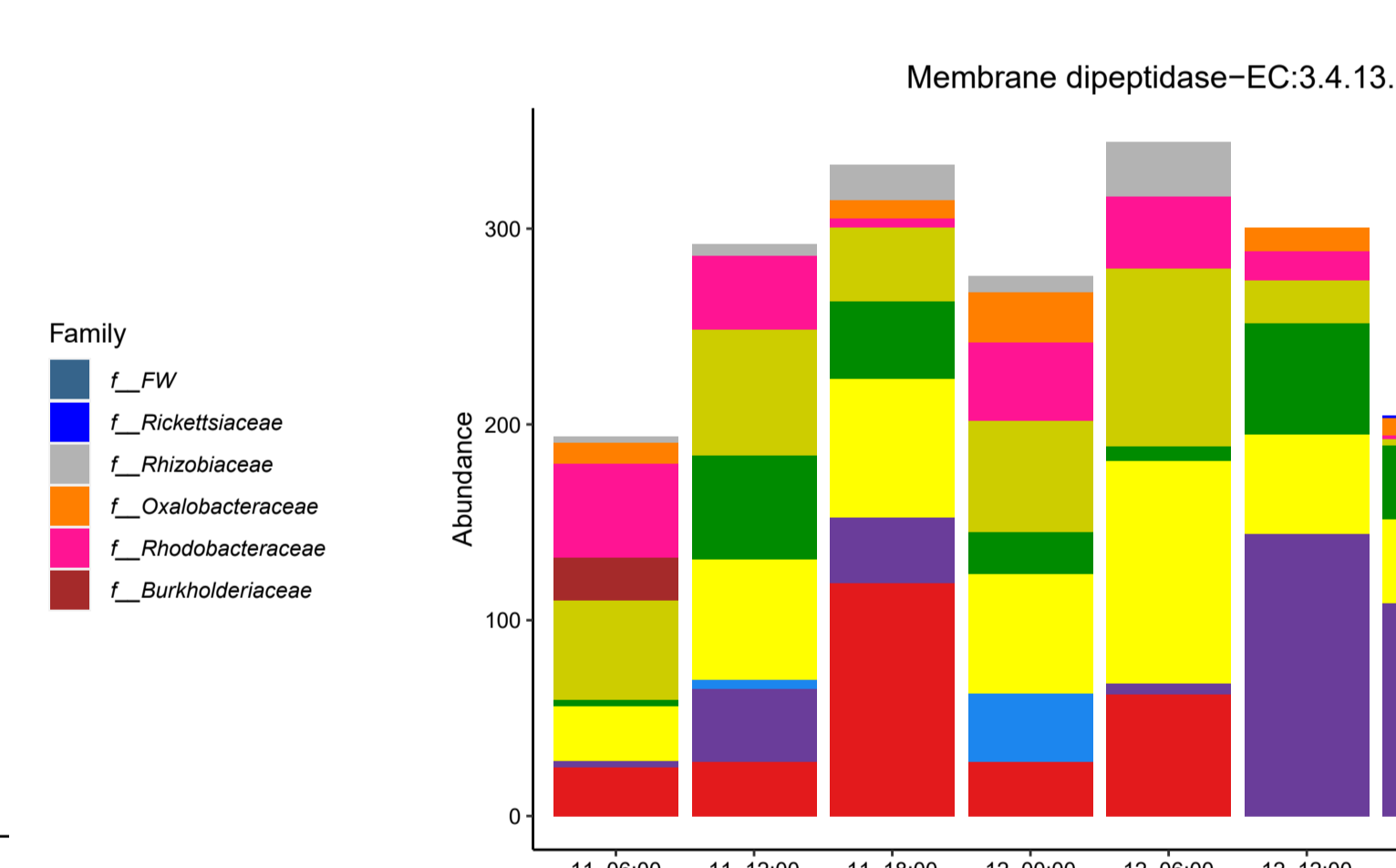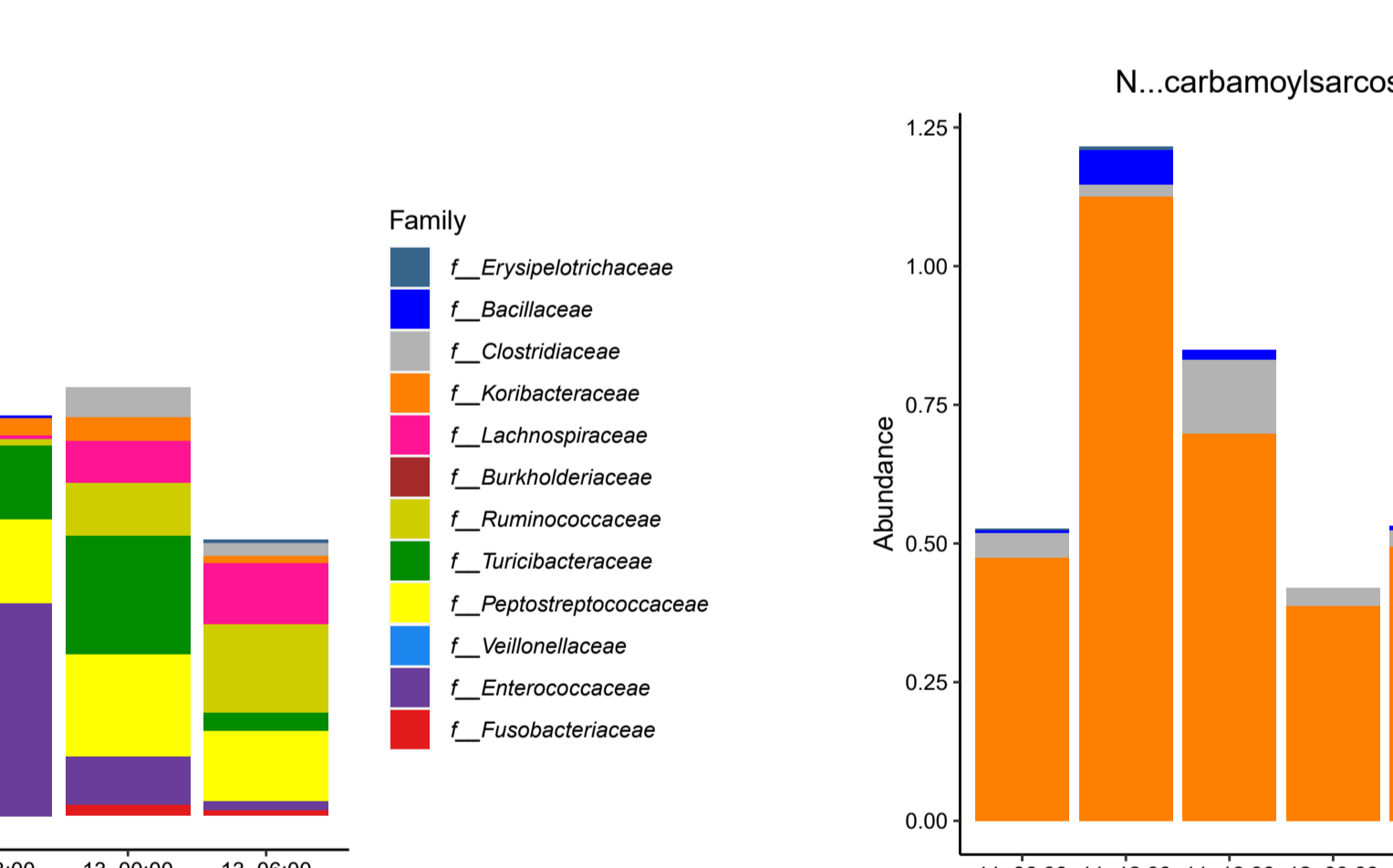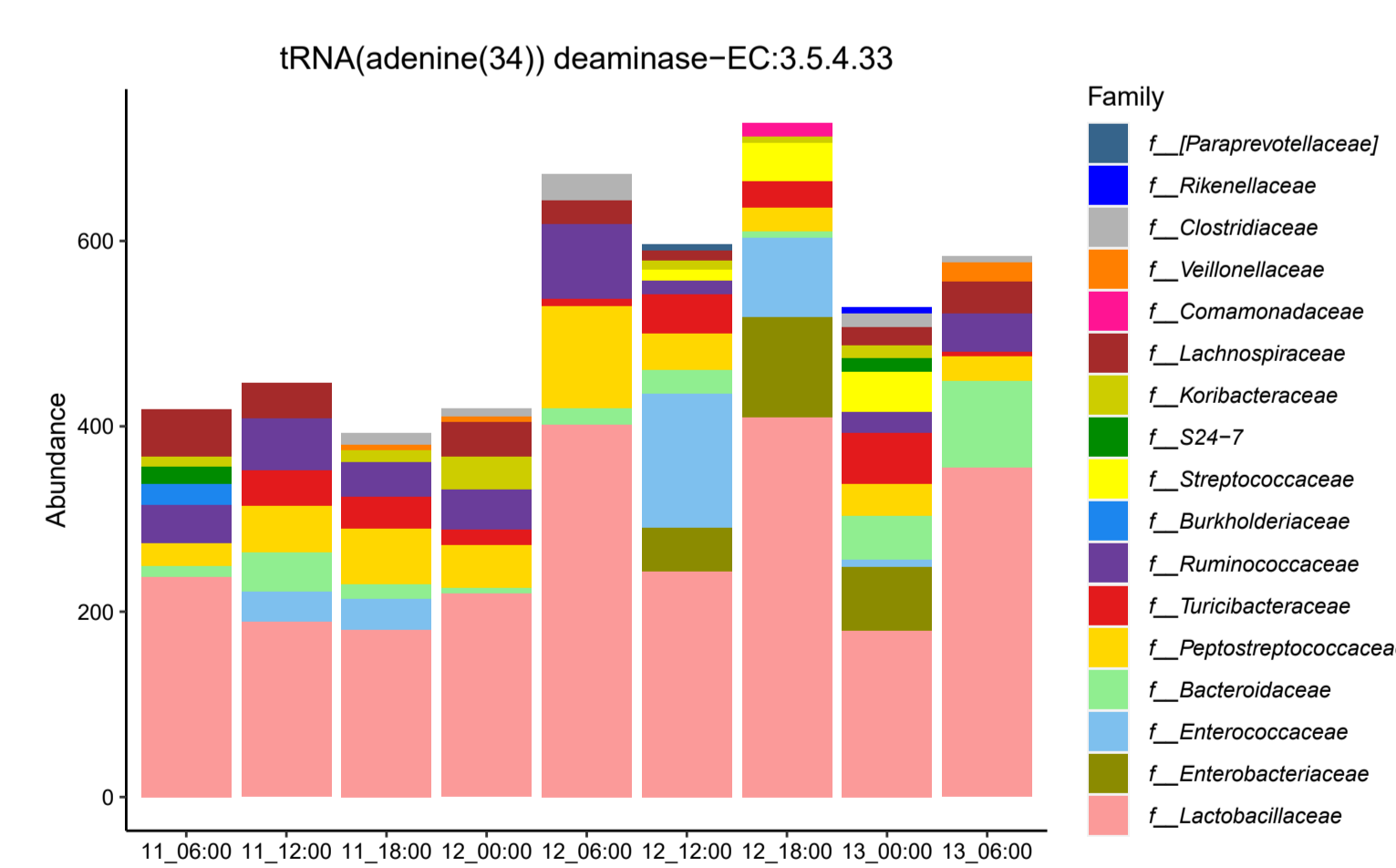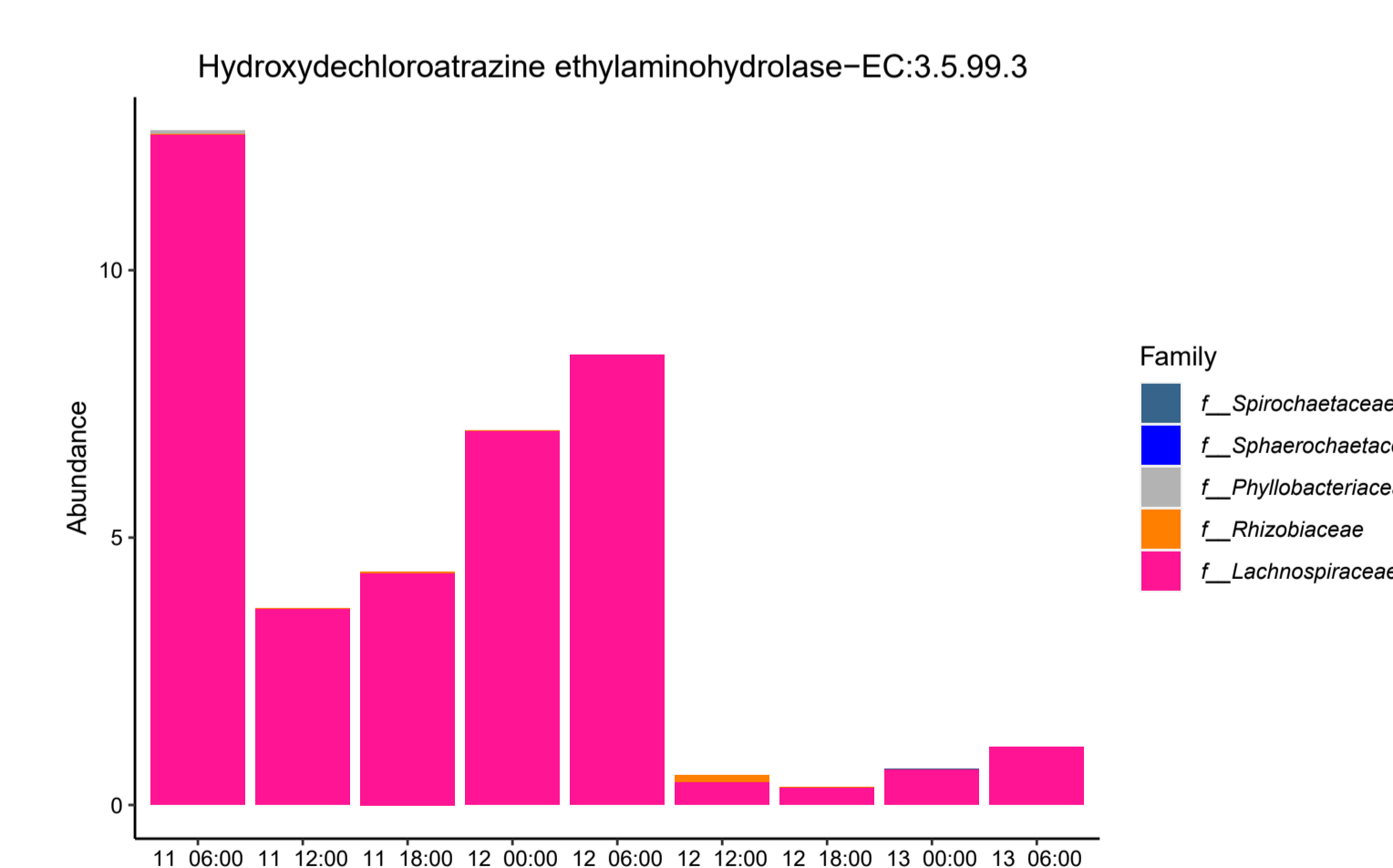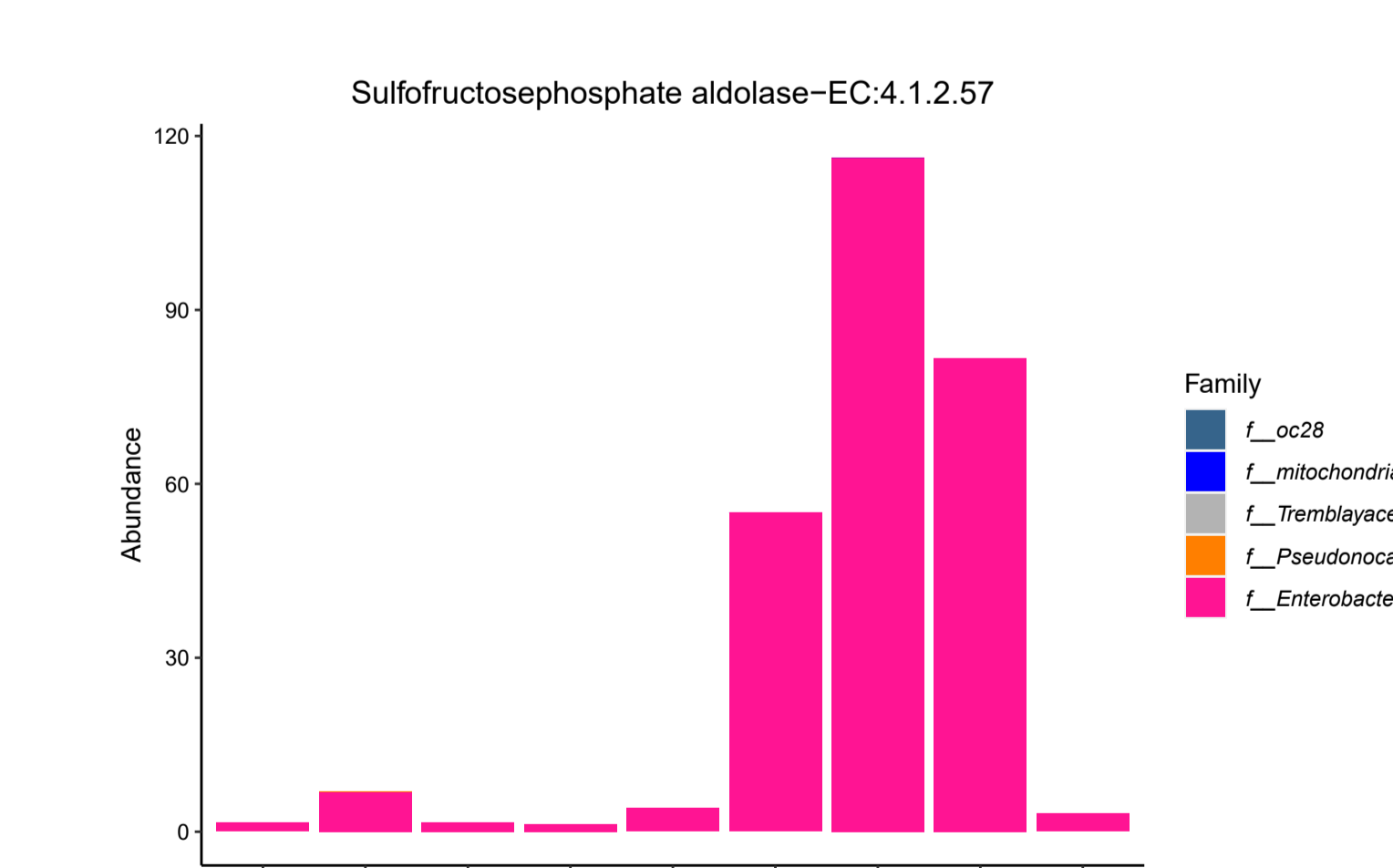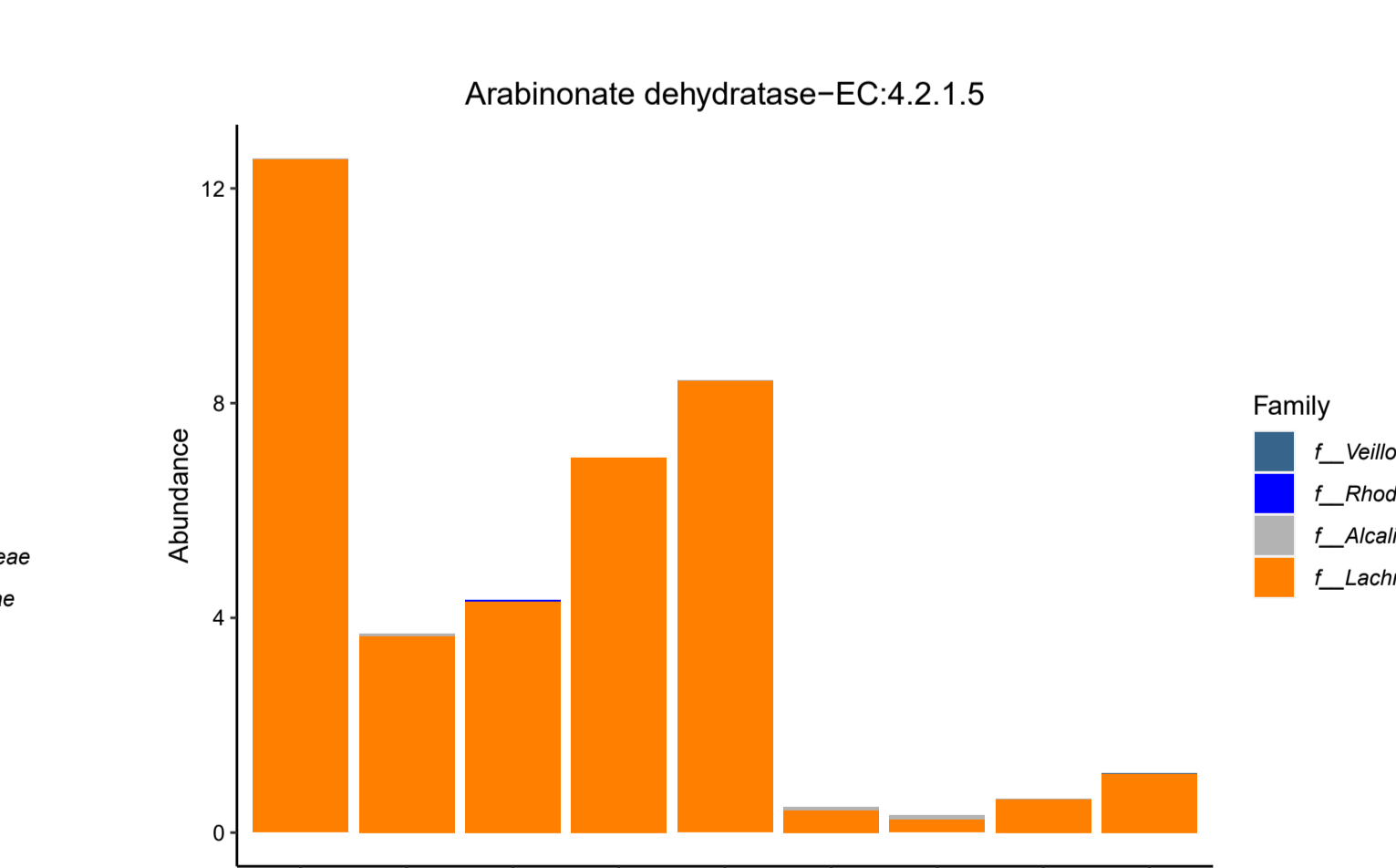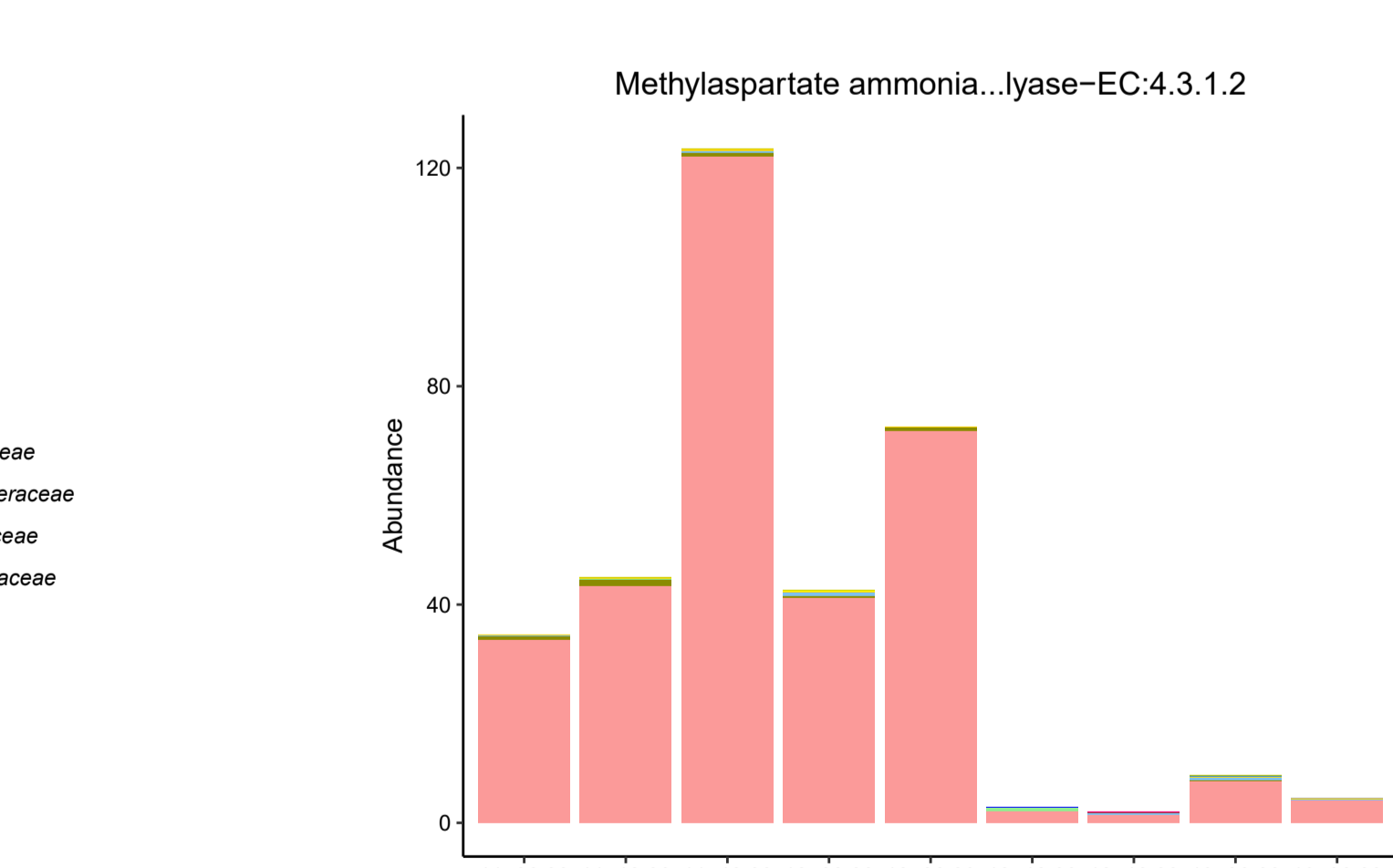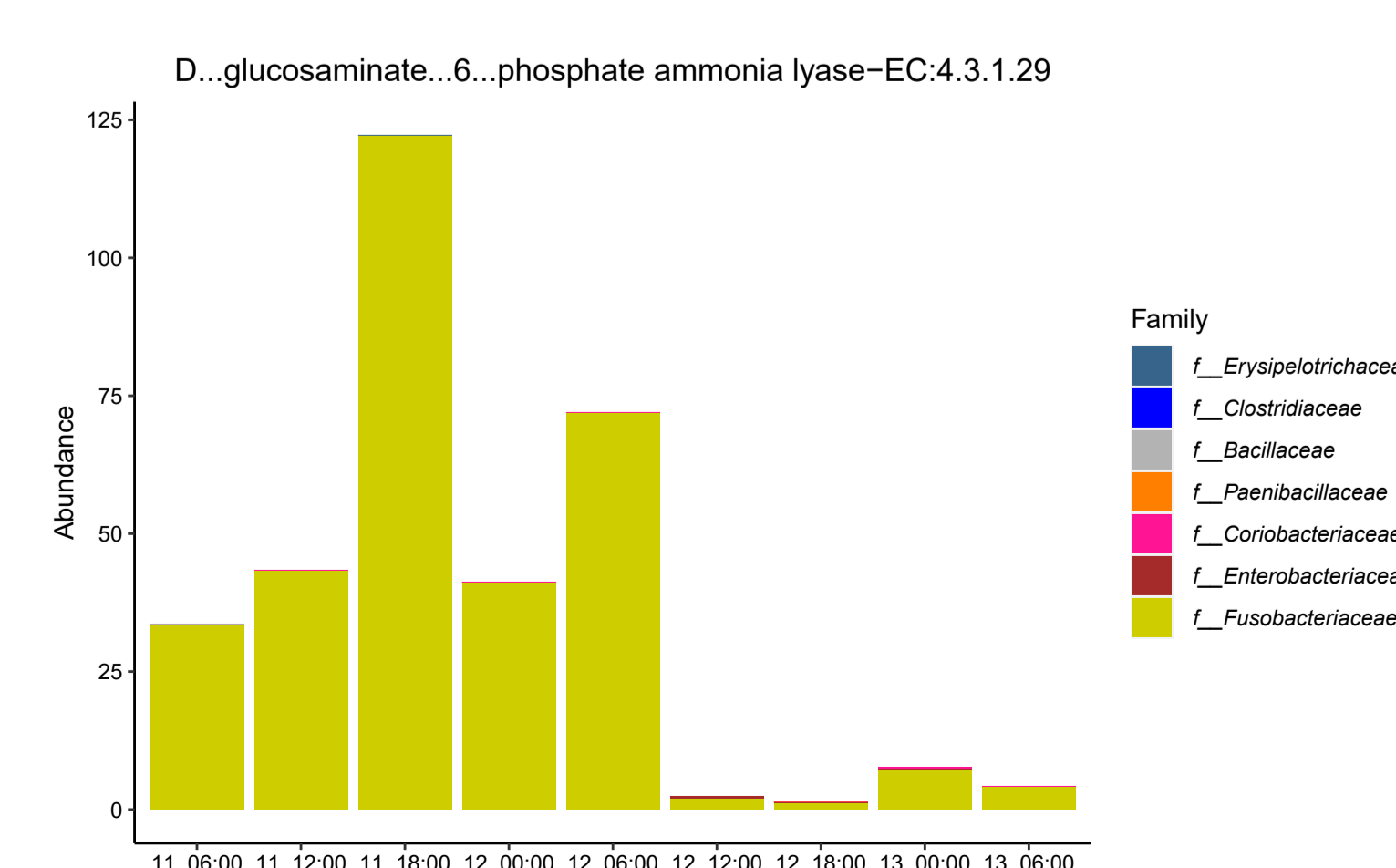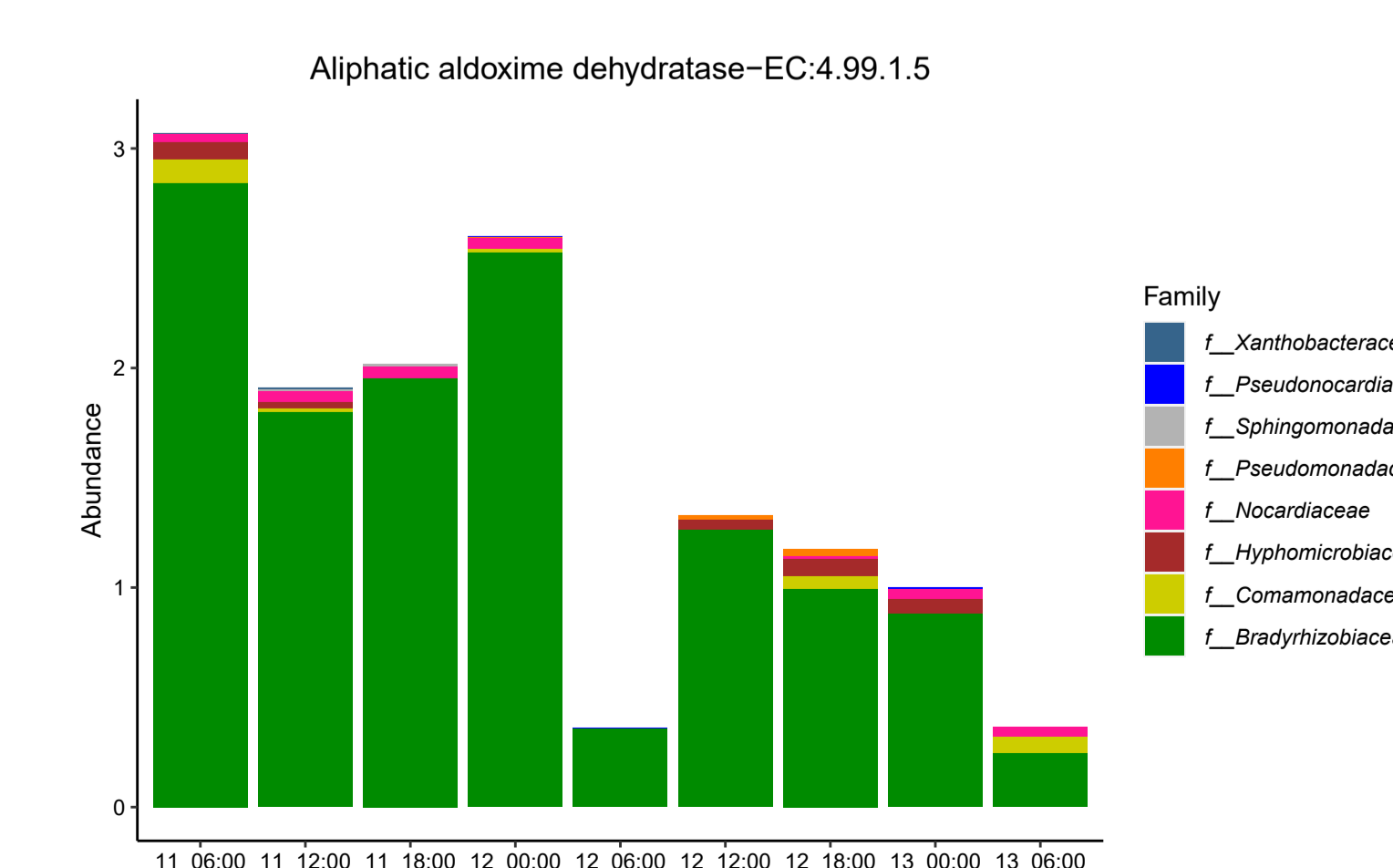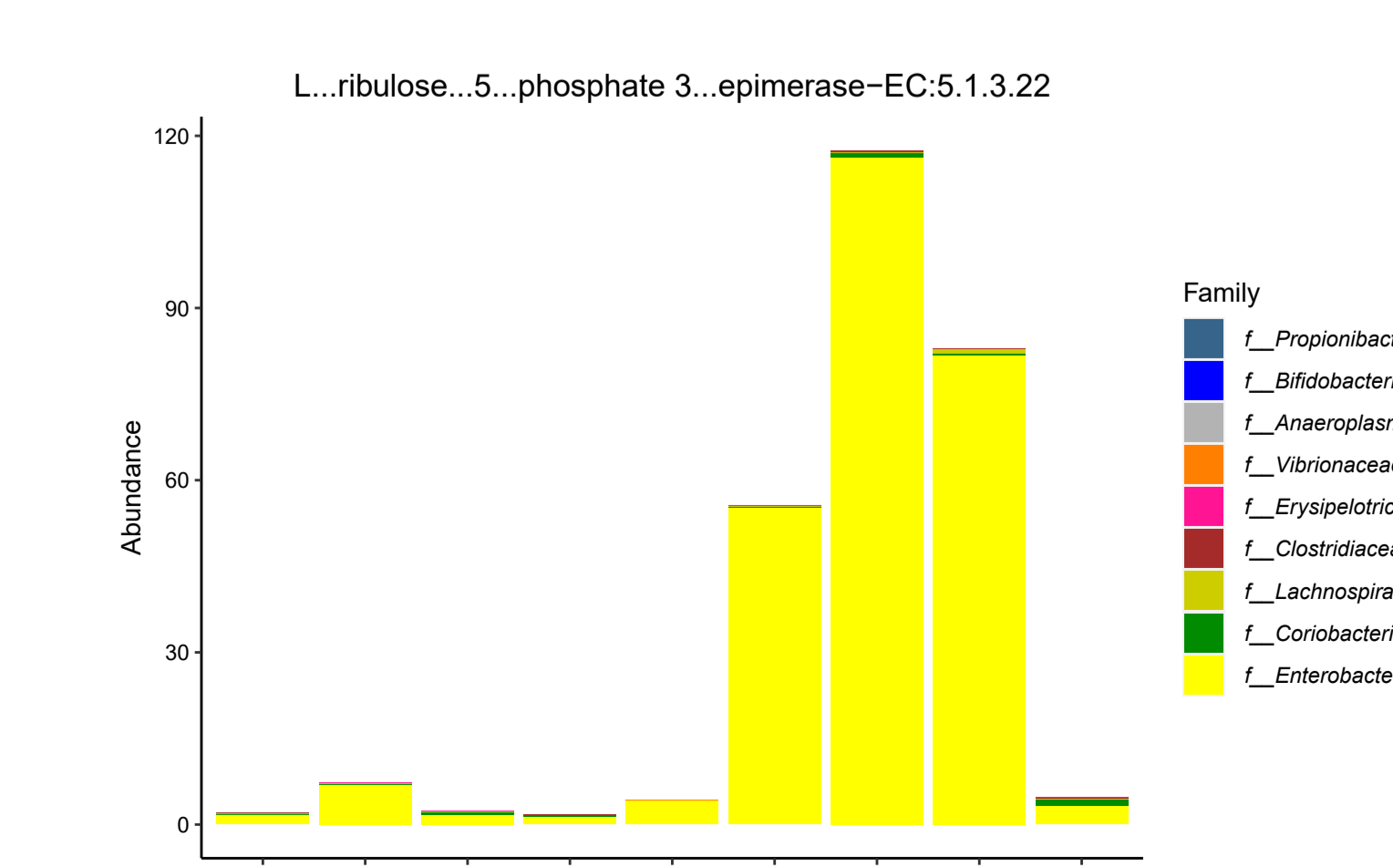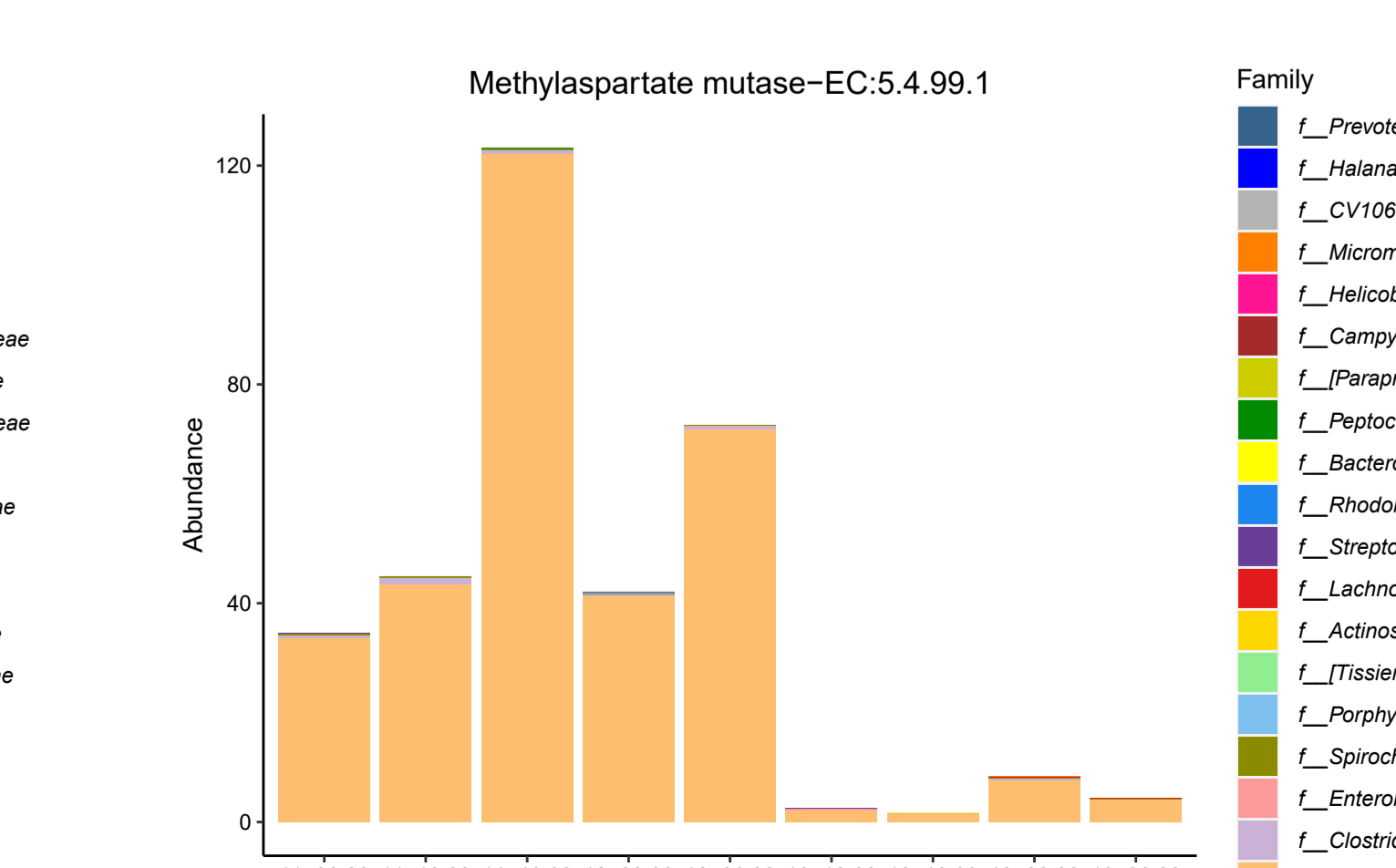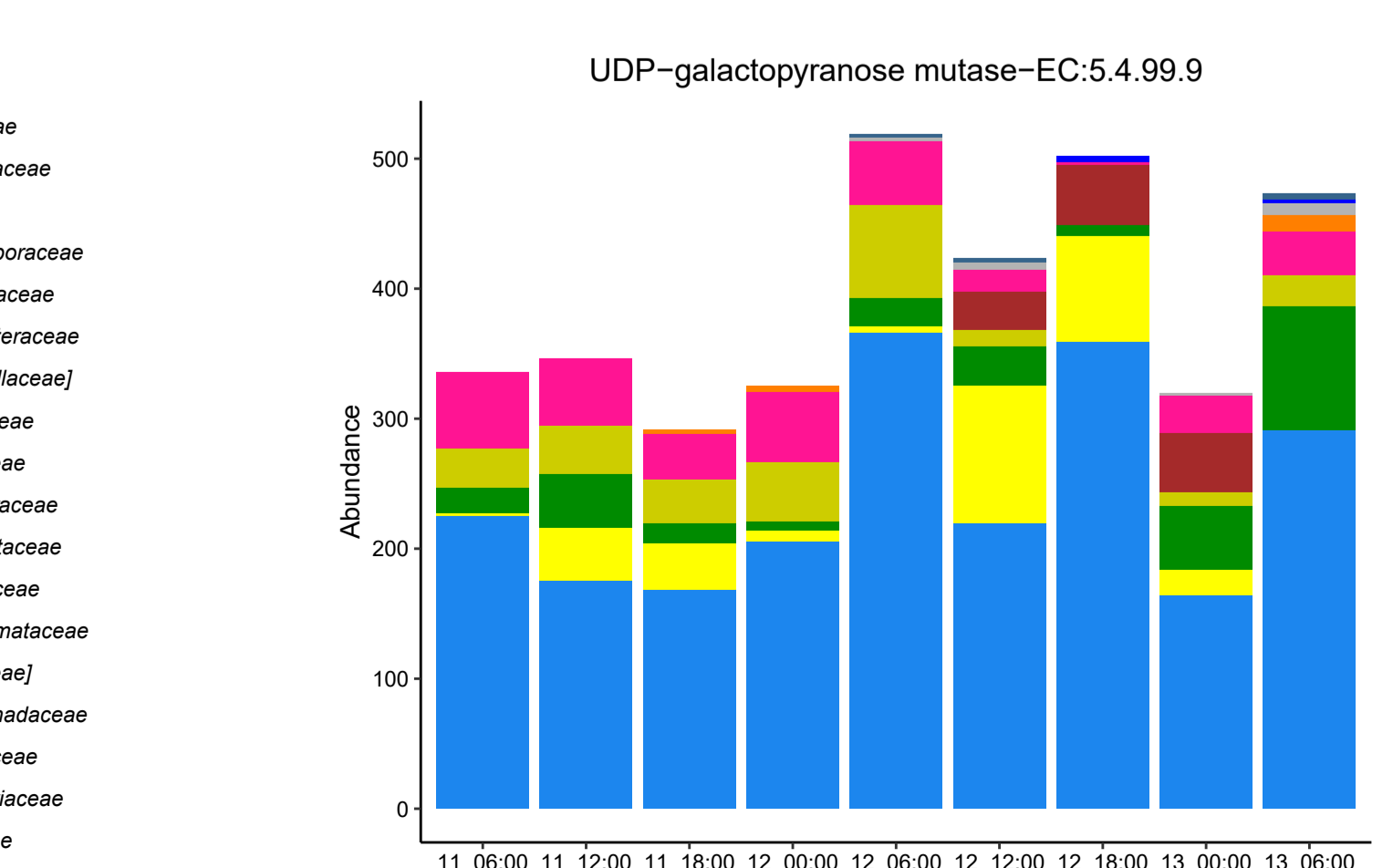

Supplement: Supplementary file 1 [file animals-11-02065-s001.zip › animals-1263808-supplementary/Figure S4.pdf]

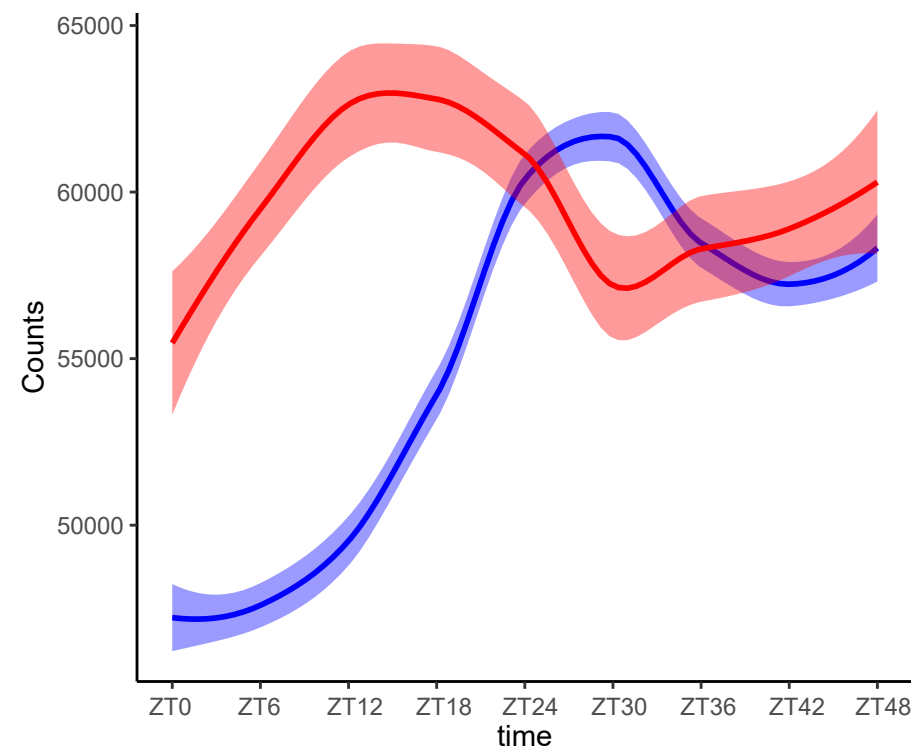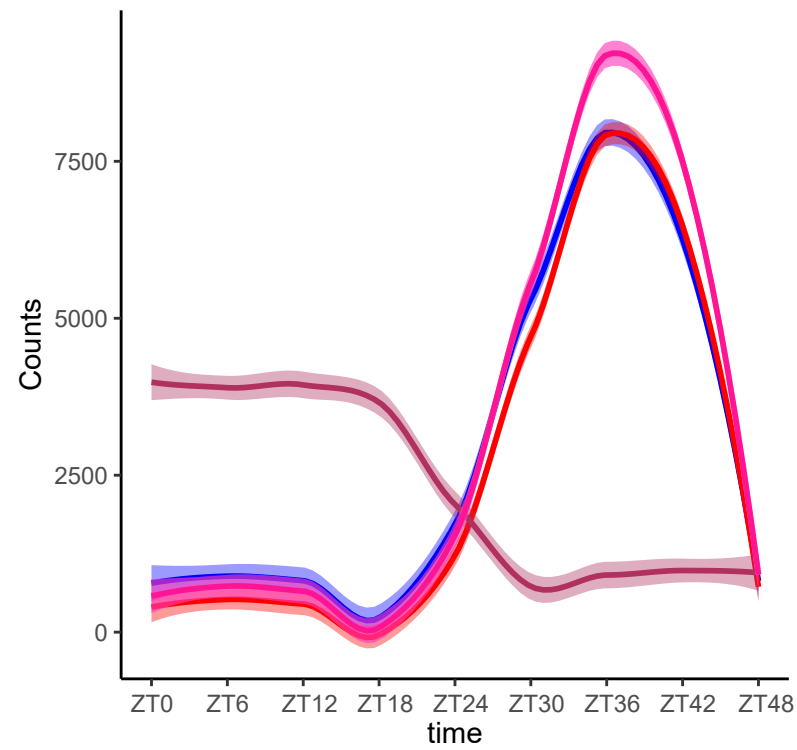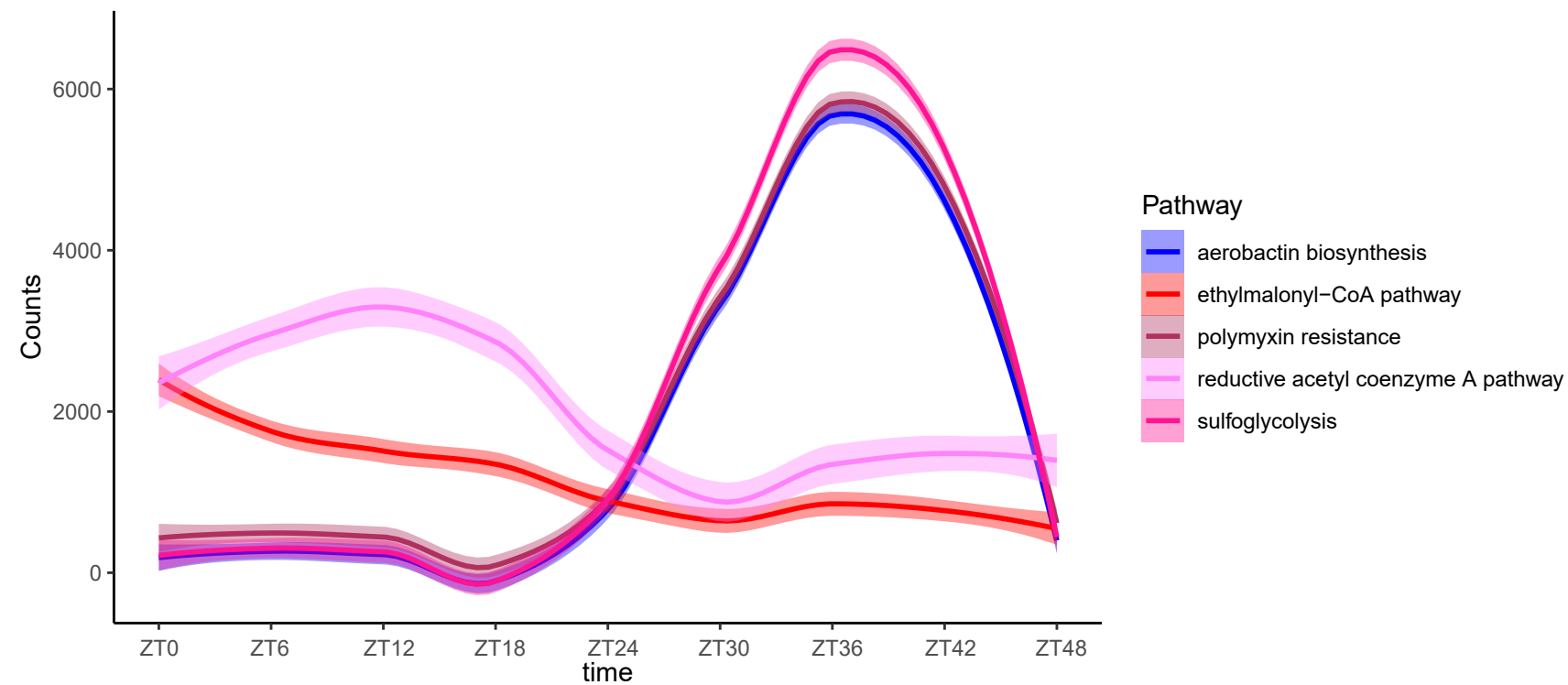

Supplement: Supplementary file 1 [file animals-11-02065-s001.zip › animals-1263808-supplementary/Figure S5.pdf]
